# Docking of acetyl-CoA carboxylase to the plastid envelope membrane attenuates fatty acid production in plants

**DOI:** 10.1038/s41467-020-20014-5

**Published:** 2020-12-03

**Authors:** Yajin Ye, Krisztina Nikovics, Alexandra To, Loïc Lepiniec, Eric T. Fedosejevs, Steven R. Van Doren, Sébastien Baud, Jay J. Thelen

**Affiliations:** 1 Department of Biochemistry, Christopher S. Bond Life Sciences Center, University of Missouri-Columbia, 1201 E Rollins, Columbia, MO 65211 USA; 2 Institut Jean-Pierre Bourgin, INRAE, CNRS, AgroParisTech, Université Paris-Saclay, 78000 Versailles, France

**Keywords:** Biofuels, Light responses, Plant biotechnology, Secondary metabolism

## Abstract

In plants, light-dependent activation of de novo fatty acid synthesis (FAS) is partially mediated by acetyl-CoA carboxylase (ACCase), the first committed step for this pathway. However, it is not fully understood how plants control light-dependent FAS regulation to meet the cellular demand for acyl chains. We report here the identification of a gene family encoding for three small plastidial proteins of the envelope membrane that interact with the α-carboxyltransferase (α-CT) subunit of ACCase and participate in an original mechanism restraining FAS in the light. Light enhances the interaction between carboxyltransferase interactors (CTIs) and α-CT, which in turn attenuates carbon flux into FAS. Knockouts for CTI exhibit higher rates of FAS and marked increase in absolute triacylglycerol levels in leaves, more than 4-fold higher than in wild-type plants. Furthermore, WRINKLED1, a master transcriptional regulator of FAS, positively regulates *CTI1* expression by direct binding to its promoter. This study reveals that in addition to light-dependent activation, “envelope docking” of ACCase permits fine-tuning of fatty acid supply during the plant life cycle.

## Abstract (web summary)

In plants, light-dependent activation fatty acid synthesis (FAS) is mediated in part by acetyl-CoA carboxylase (ACCase). Here the authors identify a family of genes encoding carboxyltransferase interactors that attenuate FAS in the light by docking acetyl-CoA carboxylase to the plastid envelope.

## Introduction

Vegetable oils are an important source of food, renewable energy, and industrial feedstocks. As demand for this commodity steadily increases, development of improved oil crops has become an agronomic priority^1^. Oil biosynthesis begins with the de novo fatty acid synthesis (FAS) pathway in the plastids. The acyl chains thus obtained are eventually esterified to glycerol to produce triacylglycerols (TAGs), the major form of storage lipid in plants^1^. Like many biosynthetic processes in plastids, the overall rate of FAS oscillates with light/dark cycles, increasing in the light and decreasing in the dark. Upon light illumination, stromal pH, Mg^2+^, ATP/ADP ratio, and redox potential increase in the chloroplasts, promoting carbon fixation and FAS^2,3^. Light-dependent control of FAS is partially achieved by regulation of acetyl-coenzyme A carboxylase (ACCase; EC.6.4.1.2) which catalyzes the first committed step in FAS, the ATP-dependent formation of malonyl-CoA from acetyl-CoA and bicarbonate^4,5^. In the plastids of dicots and non-graminaceous monocots, the predominant form of ACCase is a multisubunit, heteromeric complex (htACCase) comprised of four distinct subunits named biotin carboxylase (BC), biotin carboxyl carrier protein (BCCP), and α- and β-carboxyltransferases (CTs). In vitro, increasing pH, Mg^2+^, ATP/ADP ratio, as well as reducing conditions activates ACCase^4,5^. Additionally, ACCase is subject to feedback inhibition by downstream metabolites^6,7^ and effector proteins like the PII protein, a signal integrator that interacts with BCCP subunits to reduce the Vmax of the enzyme^8^. Additionally, a set of noncatalytic biotin/lipoyl attachment domain-containing (BADC) proteins are involved in the regulation of ACCase activity, including light-dark modulation^9–11^.

Here, we report the isolation and characterization of a gene family, *CARBOXYLTRANSFERASE INTERACTORs* (*CTIs*), encoding three plastid membrane proteins, which are involved in the regulation of de novo FAS. Using the noncatalytic domain of α-CT as bait in a yeast two-hybrid screen, we identified *CTI1*, an uncharacterized gene. The *cti* mutants display higher rates of FAS and higher TAG content in leaves. Expression of *CTI1* is activated by the WRINKLED1 transcription factor. Aside from its overall activating role in FAS, light can simultaneously attenuate FAS by promoting interactions between α-CT and CTIs, which in-turn inhibit htACCase activity and subsequently fatty acid synthesis. Our results provide important insights into how plants control light-dependent FAS to meet the cellular demand for acyl chains.

## Results

### The noncatalytic domain of α-CT directly interacts with CTIs

In seed plants, the α-CT subunit of htACCase contains a large, noncatalytic C-terminus predicted to have coiled-coil structure, a structural motif that typically mediates protein-protein interactions^12^. This observation prompted us to identify potential interactors with the putative coiled-coil domain of α-CT. Using the C-terminus (amino acid 420–769) of *Arabidopsis thaliana* α-CT as bait, a yeast two-hybrid (Y2H) screen was performed. Among 16 candidates, the screen identified the C-terminus of At1g42960 from two different clones. This candidate was named CARBOXYLTRANSFERASE INTERACTOR1 (CTI1). The sequence of the CTI1 protein contains a chloroplast transit peptide, a single transmembrane domain, followed by a coiled-coil domain (Supplementary Fig. 1a–d and Supplementary Table 1). By surveying the Arabidopsis genome, we found two *CTI1* homologs, *CTI2* (At3g02900) and *CTI3* (At5g16660), and the three CTIs share similar predicted protein domains (Supplementary Fig. 1a–d and Supplementary Table 1). Based on phylogenetic analysis (Supplementary Fig. 2, and Supplementary Table 2), the CTI family appears to be of cyanobacterial origin, with the C-terminal domain conserved between plants and cyanobacteria. While green algae, bryophytes, and gymnosperms generally possess a single *CTI*, an apparent duplication event in an ancestral angiosperm gave rise to two divergent angiosperm *CTI* subfamilies, one of which includes Arabidopsis *CTI1*, while the other includes the closely-related Arabidopsis *CTI2*/3 genes. Interestingly, the *CTI1* subfamily is apparently absent from grasses that lack the heteromeric form of ACCase. In contrast, the *CTI2/3* subfamily is conserved across major angiosperm groups.

**Fig. 1:**
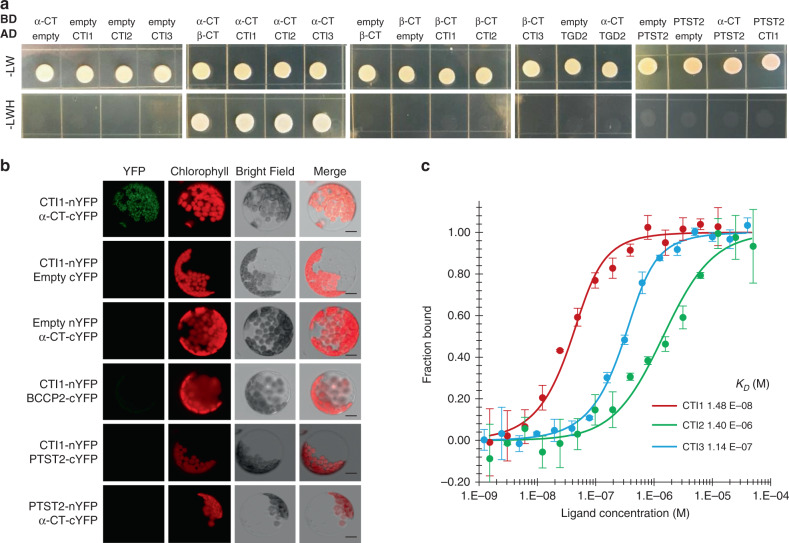
CTIs physically interact with α-CT in vitro and in vivo. **a** Yeast two-hybrid assay testing interactions between pairs of proteins. Each protein tested was fused to a GAL4 DNA-binding domain (BD) or to a GAL4 activation domain (AD). Representative cells grown on synthetic dropout (SD)-LW or SD-LWH medium with 1 mM 3-amino-1,2,4-triazole are presented. The experiment was repeated three times with similar results. **b** BiFC assays. Experiments were repeated three times with similar results. For each of the three independent protoplast transformations, at least three different protoplasts were observed. Bars = 10 μm. **c** Microscale thermophoresis (MST). Data points indicate the fraction of labeled α-CT coiled-coil domain bound to CTIs. Dissociation constants (*K*_*D*_), expressed in M, are presented. Data points are means ± SD, *n* = 3 individual biological replicates.

To confirm the interaction between α-CT and CTI1, we cloned the coiled-coil-containing C-terminus of CTI1 (Supplementary Fig. 1a) and full-length α-CT for targeted Y2H assays. The results confirmed an interaction of α-CT with CTI1, as well as with the CTI2 and 3 proteins (Fig. 1a). To control for assay specificity, the interactions between CTIs and β-CT, TRIGALACTOSYLDIACYLGLYCEROL2 (TGD2)^13^ or the coil-coiled-containing PROTEIN TARGETING TO STARCH2 (PTST2)^14^ with α-CT were tested; the results showed no interactions between either of these pairs (Fig. 1a). Next, we conducted bimolecular fluorescence complementation (BiFC) assays using a split YFP experimental system in Arabidopsis protoplasts. When *CTI1-nYFP* and α*-CT-cYFP* were coexpressed in protoplasts, a YFP signal was detectable that colocalized with chlorophyll, confirming α-CT/CTI1 interaction in planta (Fig. 1b). In contrast, no interaction could be detected between CTI1 and BCCP2 or PTST2. Additionally, both CTI2 and 3 interacted with α-CT in BiFC assays (Supplementary Fig. 3a, b). Binding affinity of the CTIs for α-CT was then quantified by microscale thermophoresis (MST) using the C-terminal sequence of both CTIs and α-CT. CTI1 exhibited a 10- and 100-fold higher affinity for α-CT compared to CTI3 and 2, respectively, based upon *K*_*D*_ values (Fig. 1c). We thus identified a family of proteins that associate with htACCase through direct interaction with α-CT.

### CTI proteins are localized in the chloroplast inner envelope membrane

**Fig. 2:**
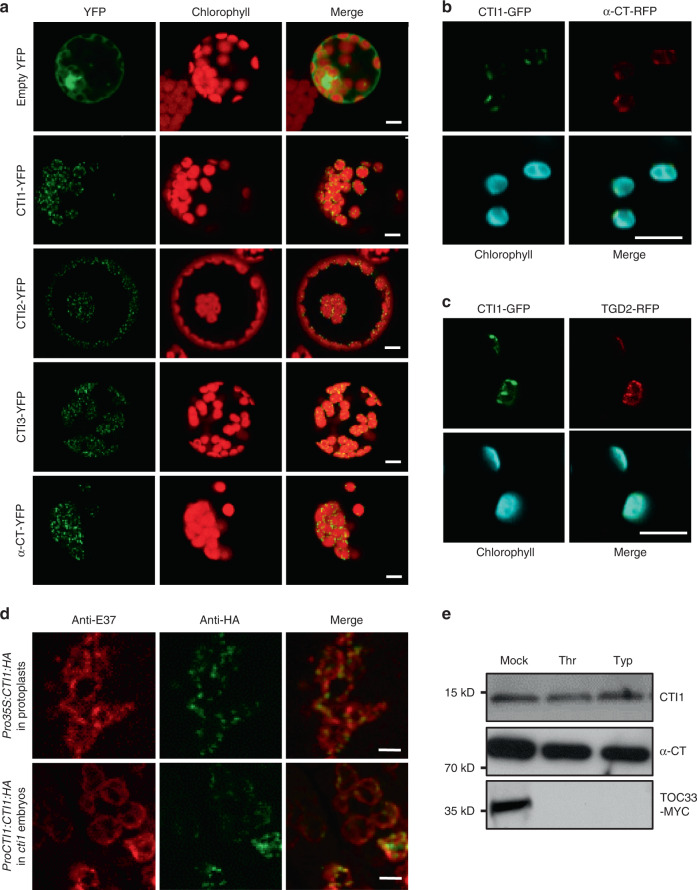
CTIs are localized in the inner envelope membrane of chloroplasts. **a** Localization of CTIs and α-CT in Arabidopsis protoplasts. CTIs and α-CT were fused with YFP, and expressed in protoplasts. The fluorescence was detected by confocal microscopy 16 h after transformation. Empty vector was used as a control. Experiments were repeated three times with similar results. For each of the three independent protoplast transformations, at least five different protoplasts were observed. Bars = 5 µm. **b** Colocalization of CTI1-GFP and α-CT-RFP fusions transiently coexpressed in leaves of *Nicotiana benthamiana* and observed two days after infiltration. Experiments were repeated three times with similar results. For each of the three independent transformations, at least five different cells were observed. Bars = 5 µm. **c** Colocalization of CTI1-GFP and TGD2-RFP fusions coexpressed in leaves of *Nicotiana benthamiana* and observed two days after infiltration. Experiments were repeated three times with similar results. For each of the three independent transformations, at least five different cells were observed. Bars = 5 µm. **d** Co-immunolocalization of CTI1 and the E37 marker of the inner envelope membrane of chloroplasts. Co-immunolocalization experiments were carried out in using anti-E37 primary antibodies and secondary antibodies conjugated to Alexa Fluor 568 (fluorescence observed at 603-682 nm; red signal) or specific anti-HA antibodies and secondary antibodies conjugated to Alexa Fluor 488 (fluorescence observed at 495–585 nm; green signal). Merged pictures are also presented. Experiments with protoplasts were repeated three times with similar results. For each of the three independent protoplast transformations, eight different protoplasts were observed. Experiments with embryos were repeated twice with similar results. In each experiment, 10 different cells from distinct embryos were observed. Bars = 5 µm. **e** Topology of the CTI1 protein. Intact Arabidopsis chloroplasts were digested by thermolysin (Thr) or trypsin (Typ) for 30 min. Proteins were detected with anti-CTI1 or anti-α-CT antibodies. TOC33-MYC fusion protein, used as a control, was detected with anti-MYC antibodies. The results shown are representative of three biologically independent samples.

Consistent with in silico predictions, CTIs were previously detected in chloroplast envelope membranes^15^. Transient expression of constructs encoding for CTI-YFP fusion proteins in Arabidopsis protoplasts revealed discrete foci at the periphery of chloroplasts similar to that observed with α-CT-YFP (Fig. 2a) and TGD2-GFP fusions^13^. This pattern of localization also mirrored the distribution of two other ACCase interactors, i.e., PII and BADC1 proteins, in plastids^10,16^. It is not clear, however, whether or not these foci denote subdomains of the membrane where htACCase units are preferentially clustered in a wild-type context, considering that protein overexpression produced similar patterns^17^. To specifically confirm colocalization of CTI1 with TGD2, a well-characterized protein of the plastid inner envelope membrane^13^, or with the CT subcomplex of htACCase previously shown to be associated with the plastid inner envelope membrane^18–20^, *CTI1-GFP* was transiently coexpressed with α*-CT-RFP* (Fig. 2b) or with *TGD2-RFP* (Fig. 2c) in leaves of *Nicotiana benthamiana*. Colocalization of GFP and RFP signals suggested that CTI1 is indeed a component of the chloroplast inner envelope membrane. Furthermore, co-immunolocalization experiments were carried out between CTI1 and E37, a methyltransferase involved in tocopherol and plastoquinone synthesis localized to the plastid inner envelope membrane^21–23^. In Arabidopsis protoplasts transfected with the *Pro35S:CTI1:HA* construct and in embryonic cells of Arabidopsis transgenic lines stably transformed with the *ProCTI1:CTI1:HA* construct, the CTI1:HA fusion protein appeared as a punctate pattern colocalized with a subset of the wider distribution of E37, confirming targeting of CTI1 to the chloroplast inner envelope membrane (Fig. 2d). Finally, the membrane topology of CTI1 was determined by dual-protease digestion assay. Intact chloroplasts were isolated from Arabidopsis leaf tissues and treated with thermolysin (a protease unable to penetrate the outer envelope membrane) or trypsin (a protease able to penetrate the outer envelope but not the inner envelope membrane). CTI1, like α-CT, was resistant to both proteases, suggesting that the C-terminus of the CTI1 protein faces the stromal side of the inner envelope membrane (Fig. 2e).

### CTIs negatively regulate fatty acid synthesis through htACCase

**Fig. 3:**
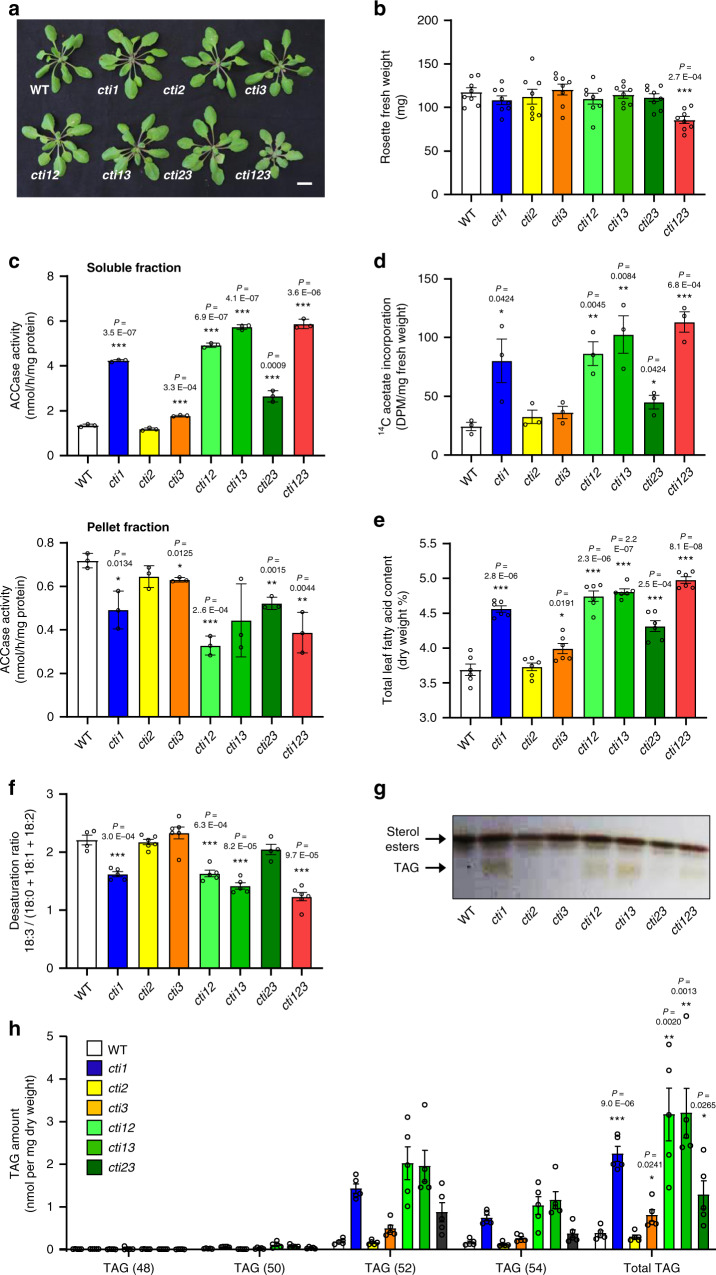
CTIs negatively regulate fatty acid synthesis through htACCase. **a** 4-week old plants grown in long-day (LD) conditions. **b** Above-ground biomass of 4-week-old plants. Data are means ± SEM, *n* = 8 individual biological replicates. Asterisks indicate significant difference from the wild-type control as determined by a two-tailed Student’s *t*-test at ***P < 0.001. **c** htACCase activity in the soluble and pellet fractions of crude chloroplasts from 4-week-old rosette leaves. Data are means ± SEM, *n* = 3 individual biological replicates. Asterisks indicate a significant difference from the wild-type control as determined by a two-tailed Student’s *t-*test at ***P < 0.001. **d** Rates of fatty acid synthesis in detached rosettes leaves estimated thanks to the incorporation of labeled acetate. Data are means ± SEM, *n* = 3 biologically independent plants. Asterisks indicate significant difference from the wild-type control as determined by a two-tailed Student’s *t*-test at ***P < 0.001, **P < 0.01, and *P < 0.05, respectively. **e** Leaf total fatty acid content determined by gas chromatography. Data are means ± SEM, *n* = 6 individual biological replicates. Asterisks indicate significant difference from the wild-type control as determined by a two-tailed Student’s *t*-test at ***P < 0.001, and *P < 0.05, respectively. **f** Desaturation ratio among C18 fatty acids in rosette leaves. Data are means ± SEM, scatterplots show individual biological replicates as circles. Asterisks indicate significant difference from the wild-type control as determined by a two-tailed Student’s *t*-test at ***P < 0.001. **g** Accumulation of triacylglycerols (TAG) in rosette leaves of *cti* mutants observed by thin-layer chromatography. Experiments were repeated three times with similar results. **h** Triacylglycerol (TAG) composition of rosette leaves as determined by ESI-tandem mass spectrometry. The numbers between brackets indicate the number of carbon atoms of the TAG species analyzed. Data are means ± SEM, *n* = 5 biologically independent plants. Asterisks indicate significant difference from the wild-type control as determined by a two-tailed Student’s *t-*test at ***P < 0.001, **P < 0.01, and *P < 0.05, respectively.

To determine the function of *CTIs* in Arabidopsis, we used CRISPR/Cas9 to knockout the *CTI* genes individually. We recovered CRISPR/Cas9-induced homozygous frameshift mutants for each *CTI* gene (Supplementary Fig. 4a). The frameshifts induced premature termination codons preceding the coiled-coil domain for all three CTIs (Supplementary Fig. 4b). The efficacy of the *cti1* knockout mutant was confirmed by immunoblotting using anti-CTI1 antibody (Supplementary Fig. 4c). Double and triple mutants were then generated by crosses to examine genetic interactions among the *CTIs*. When grown under long-day conditions, the *cti123* triple mutant displayed a growth defect with smaller rosette leaves and decreased aerial biomass compared to single and double mutants or wild-type (WT) plants, indicating a growth penalty in the triple mutant (Fig. 3a, b).

We then assayed ACCase activity in leaves of the different genotypes. Soluble protein fractions from crude chloroplasts prepared from wild-type leaves were assayed in the presence of two different ACCase inhibitors; avidin, a general inhibitor of ACCase activity, and the herbicide haloxyfop, which specifically targets homomeric ACCase^24^. Whereas addition of avidin strongly reduced the measured ACCase activity, haloxyfop did not cause a significant reduction in specific activity (Supplementary Fig. 5), demonstrating that htACCase activity predominates in the plant material prepared. Then, supernatants and pellets from clarified (13,000 × *g*) chloroplast lysates from 4-week-old leaves of the different *cti* mutants were assayed for soluble and membrane-bound ACCase activity, respectively. In the supernatants, the *cti1* mutation yielded a 3.5-fold increase in ACCase activity with respect to the WT control. ACCase activity was further enhanced in *cti13* and *cti123* mutants (Fig. 3c). Conversely, in the pellets, the *cti1* mutation yielded lower ACCase activity, and further reduction was observed in the *cti123* triple mutant (Fig. 3c). To estimate the rate of FAS in *cti* mutants, we performed in vivo ^14^C-acetate labeling experiments using 4-week-old leaf strips. After an incubation of 40 min, the rate of ^14^C-acetate incorporation was around 3.5-fold higher in *cti1* than in WT, while a slight increase was observed in *cti3* mutant. The double and triple mutants exhibited enhanced rates of incorporation with respect to the single mutants (Fig. 3d), reflecting the variations observed in soluble ACCase activity (Fig. 3c). Incorporation of ^14^C-malonate into nascent fatty acids was not higher in *cti* mutants (Supplementary Fig. 6). Next, we quantified htACCase subunit levels in the *cti* mutants by western blotting. The results indicated that higher FAS rates were not the consequence of increased levels of htACCase subunits (Supplementary Fig. 7).

As a first approach to study the impact of higher soluble htACCase activity in *cti* mutants, we determined the total fatty acid content in leaves of plants grown under long-day (LD) photoperiod. Consistent with increased FAS rate, leaf fatty acid content in the *cti1* mutant was significantly higher than WT (a 22% increase). An additive effect of the *cti* mutations could be observed in double and triple mutants (Fig. 3e). The *cti* mutants also showed altered leaf fatty composition, with a decrease in the relative proportion of 18:3 polyunsaturated fatty acids and a concomitant increase in 18:0, 18:1, and 18:2 yielding a reduced desaturation ratio (Fig. 3f). Analysis of leaf lipid extracts by thin-layer chromatography (TLC) revealed accumulation of neutral lipids comigrating with TAGs in *cti* mutants (Fig. 3g). Comprehensive, quantitative lipidomic analyses confirmed specific accumulation of TAGs in *cti1* and *cti3* single mutants as well as an additive effect in double mutants (Fig. 3h). In contrast, TLC and quantitative lipidomic analyses of polar lipids showed that these classes of lipids were not affected by the *cti* mutations (Supplementary Fig. 8a, b). In mutant seeds, the total seed oil content was unmodified but a reduced desaturation ratio was observed as in leaves (Supplementary Fig. 9). To confirm the CRISPR mutant phenotypes, we then characterized a T-DNA insertion mutant corresponding to a second null allele of *CTI1* (Supplementary Fig. 10a). This T-DNA allele (*cti1-2*) phenocopied the *cti1* CRISPR/Cas9 allele, including increased soluble ACCase activity, FAS rate, total fatty acid content, and TAG accumulation in leaves (Supplementary Fig. 10b–f), as well as modified fatty acid composition of leaves and mature dry seeds (Supplementary Fig. 10g). This last phenotype was complemented by introgression of the *ProCTI1:CTI1:HA* or *ProAT2S2:CTI1* construct in the *cti1-2* mutant background (Supplementary Fig. 10g). A partial complementation of the *cti1-2* seed oil phenotype could also be obtained by introgression of *CTI2* and *CTI3* cDNAs placed under the control of the seed-specific *ProAT2S2* promoter^25^, but the efficiency of these phenotypic reversions was lower (Supplementary Fig. 10g). Taken together, these results demonstrate that CTIs attenuate soluble htACCase activity, so that *cti* mutations enhance carbon flux into fatty acid synthesis, resulting in TAG over accumulation in leaves. If the three CTIs have partially redundant functions, CTI1 appears to be the predominant effector of htACCase activity. The phenotypic impact of the CTI1 isoform is consistent with both higher affinity for α-CT and phylogeny data showing that CTI1 coevolved with htACCase.

### WRINKLED1 activates *CTI1* expression through direct binding to *CTI1* promoter

**Fig. 4:**
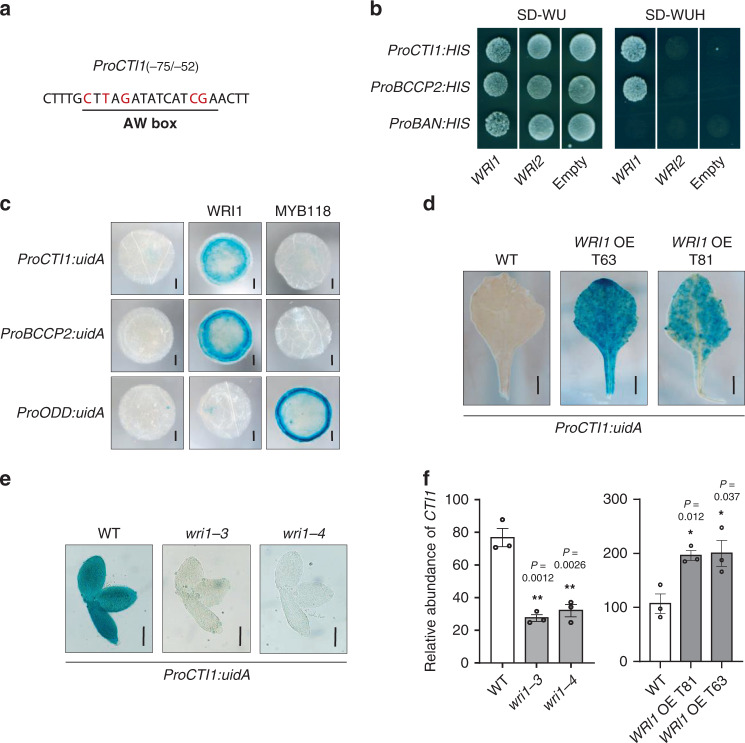
WRINKLED1 enhances *CTI1* expression through direct binding to the *CTI1* promoter. **a** AW *cis*-regulatory element present in the *CTI1* promoter. Conserved nucleotides denoting the presence of an AW box are in red. The numbers between brackets indicate the position of the element relative to the start codon. **b** WRI1 directly activates the *CTI1* promoter in yeast one-hybrid assay. Yeast strains containing the *HIS3* reporter gene under the control of either the *BANYULS* (*BAN*, negative control), *BCCP2* (positive control), or *CTI1* promoter were transformed with either the empty expression vector or with versions of the vectors allowing expression of *WRI1* or *WRI2* (negative control) before being plated on synthetic dropout (SD-WU or SD-WUH) media. The experiment was repeated five times (with independent clones) with similar results. **c** WRI1 activates the promoter of *CTI1* in transactivation assay in leaves of *Nicotiana benthamiana*. *Pro:uidA* reporter constructs (*ProBCCP2* was used as positive control, *ProODD* as negative control) alone or in combination with a vector allowing the expression of WRI1 or MYB118 (negative control) were coinfiltrated in young leaves of *N. benthamiana*. The experiment was repeated twice with similar results. For each experiment and for each condition tested, six independent infiltrations were realized. Representative pictures are displayed. Bars = 2 mm. **d** Activity of the *ProCTI1(994* *bp):uidA* cassette in rosette leaves of *Pro35Sdual:WR1* over-expressing (OE) lines (*WRI1* OE T63 and T81) and in a wild-type (WT) background. The experiment was repeated twice (using independent *ProCTI1(994* *bp):uidA* transformants) with similar results. For each experiment and for each genotype, six different leaves were observed. Representative pictures are displayed. Bars = 5 mm. **e** Activity of the *ProCTI1(994* *bp):uidA* cassette in *wri1* mutant embryos and in a WT embryo. The experiment was repeated twice (using independent *ProCTI1(994* *bp):uidA* transformants) with similar results. For each experiment and for each genotype, 10 different embryos were observed. Representative pictures are displayed. Bars = 150 µm. **f** Accumulation levels of *CTI1* transcripts in seeds of *wri1* and in leaves of two independent *WRI1* overexpressing lines (*WRI1* OE T81 and T63) analyzed by qRT-PCR. The results obtained are standardized to the constitutive *EF1*α*A4* gene expression level. Data are means ± SEM, *n* = 3 biologically independent plants. Asterisks indicate significant difference from the wild-type control as determined by a two-tailed Student’s *t-*test at **P < 0.01 and *P < 0.05, respectively.

Expression patterns of the *CTI* genes were investigated using qRT-PCR (Supplementary Fig. 11a) and translational *ProCTI:CTI-uidA* fusions assayed in transgenic plants (Supplementary Fig. 11b–d). The three *CTIs* appeared to be ubiquitously expressed, and *CTI1* mRNAs were more abundant than *CTI2* or *CTI3* mRNAs in all tissues analyzed, providing another possible explanation for the predominant regulatory role of CTI1. Furthermore, a peak of *CTI1* transcript accumulation was detected at the onset of seed maturation and this pattern was not observed for *CTI2* and *CTI3*. The expression pattern of *CTI1* in seeds was similar to the profiles observed for several genes encoding FAS enzymes known to be transcriptionally activated by the WRINKLED1 (WRI1) transcription factor, which binds AW *cis*-regulatory elements within its target promoters^25–28^. Interestingly, an AW box could be identified in the *CTI1* promoter but not in *CTI2* and *CTI3* promoters (Fig. 4a). The interaction between WRI1 and the *CTI1* promoter was demonstrated using a yeast one-hybrid approach: the expression of WRI1-AD in the strain containing the *HIS3* reporter gene under the control of the *CTI1* promoter resulted in the specific growth of the strain on medium lacking histidine (Fig. 4b). To test the ability of WRI1 to directly activate the *CTI1* promoter *in planta*, a *ProCTI1:uidA* reporter construct was then used in transactivation assays in *N. benthamiana* leaves (Fig. 4c). WRI1 was able to activate reporter constructs comprising *CTI1* or *BCCP2* promoters (positive control), showing a strong increase in β-glucuronidase (GUS) activity, but not the negative control *2-OXOGLUTARATE-DEPENDENT DIOXYGENASE* (*ODD*) promoter^29^, which could be activated by MYB118 (Fig. 4c). To further evaluate the impact of WRI1 on the pattern of *CTI1* promoter activity, the *ProCTI1:uidA* construct was stably expressed in transgenic Arabidopsis lines. Unlike *ProCTI1:CTI1:uidA* transgenic lines, *ProCTI1:uidA* lines displayed GUS staining in reproductive organs but not in vegetative parts of the plants (Supplementary Fig. 10, 11), indicating that important regulatory elements exist downstream of the translational start codon of *CTI1*. The construct was then introduced into *Pro35Sdual:WRI1* lines by crosses, and a strong induction of *ProCTI1* activity was detected in rosette leaves (Fig. 4d). In contrast to the observations made in the WT, *ProCTI1* activity could not be detected in *wri1* embryos (Fig. 4e). In agreement with these observations, *CTI1* mRNA levels were significantly decreased in *wri1* embryos, whereas higher *CTI1* mRNA levels were measured in leaves of *WRI1* overexpressors (Fig. 4f). Together, these results showed that WRI1 enhances the activity of the *CTI1* promoter, in a similar way to the activities of promoters of genes coding core FA biosynthetic enzymes and regulators of htACCase like the BADC subunits and PII^16,30^.

### CTI/α-CT module regulates fatty acid synthesis in a light-dependent manner

**Fig. 5:**
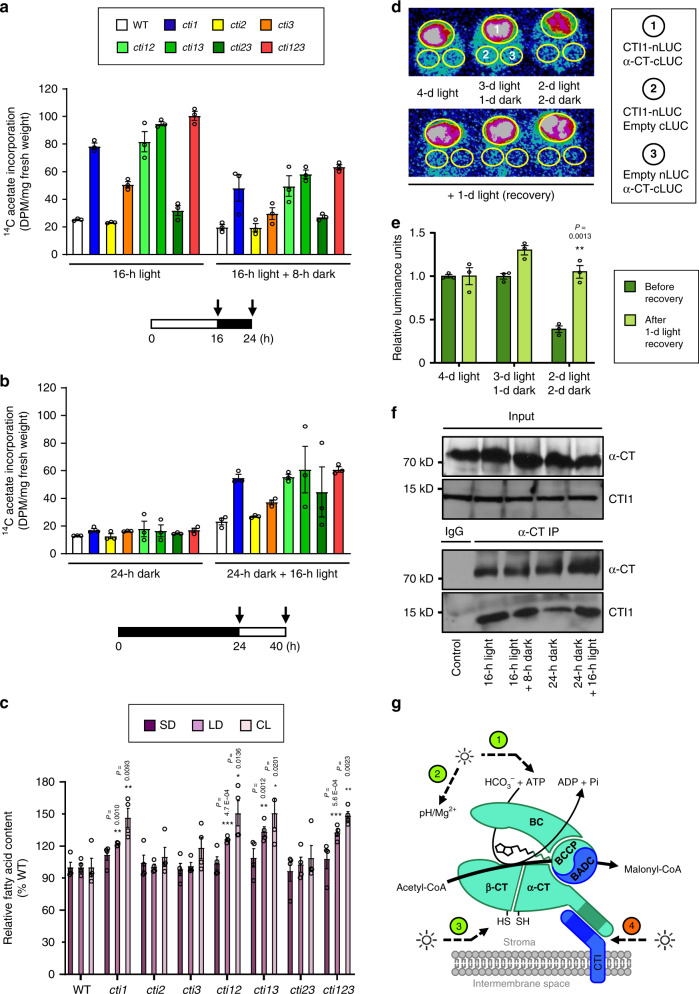
CTIs interacting with α-CT regulate fatty acid synthesis in a light-dependent manner. **a**, **b** ^14^C-acetate incorporation experiments carried out on rosette leaves aged 4 weeks and subjected to different light/dark regimes. Data are means ± SEM, *n* = 3 biologically independent plants. **c** Leaf total fatty acid content of rosette leaves from plants grown under different light regimes as determined by gas chromatography. Plants were grown in short-day (SD), long-day (LD) or constant light (CL) conditions for 4 weeks before analyses. Data are means ± SEM, *n* = 4 biologically independent plants. Asterisks indicate significant difference from the wild-type control as determined by a two-tailed Student’s *t*-test at ***P < 0.001, **P < 0.01, and *P < 0.05, respectively. **d** Split-luciferase assays. Three combinations of constructs were injected in leaves of *Nicotiana benthamiana*. After transformation, the plants were grown under different light/dark regimes before imaging of luminescence. Experiments were repeated three times with similar results. For each of the three independent transformations, three different leaves were observed. **e** Relative luminescence signal quantified after coexpression of CTI1-nLUC and α-CT-cLUC as displayed in **d**. Data are means ± SEM, *n* = 3 biologically independent plants. Asterisks indicate significant difference from the 2-d light/2d-dark treatment (before recovery) as determined by a two-tailed Student’s *t*-test at **P < 0.01. **f** Co-immunoprecipitation assays. IgG was used as a negative control. Experiments were repeated three times with similar results. **g** Model for the regulation of htACCase activity by light. Light upregulates FAS by increasing the ATP/ADP ratio (1), optimizing Mg^2+^ concentration and pH (2), and redox potential in the plastid (3). Light also attenuates htACCase activity by enhancing α-CT/CTI interactions, possibly on a different timescale (4). Additionally, BADC proteins modulate htACCase activity by competing with BCCP subunits for access to BC.

It was proposed that light-dependent ^14^C-acetate incorporation into fatty acids is due to the regulation of htACCase^31–33^. To determine if the CTIs are involved, rates of FAS in 4-week-old leaves were monitored using ^14^C-acetate labeling. Under a classical LD regime, incorporation rates were higher at the end of the day than at the end of the dark period for most genotypes. Increased ^14^C-acetate incorporation observed in *cti* mutants was moderated at the end of the dark period (Fig. 5a). The enhancement of ^14^C-acetate incorporation measured in *cti* mutants was abolished by an extended dark period (24 h) but recovered after a 16-h-light period (Fig. 5b). To exclude the possibility that the variations in incorporation rates measured were reflecting differences in unlabeled acetate in leaves, a competition assay with unlabeled acetate was performed. Before adding ^14^C-acetate, leaf tissues were incubated in the presence of unlabeled acetate for 10 min. No competition effect could be detected in the WT nor in the *cti1* mutant (Supplementary Fig. 13). However, considering that acetate is not the principal carbon source for FAS in planta^34,35^, we next quantified the total leaf fatty acid content from Arabidopsis plants grown under various photoperiods. No significant modifications of the leaf fatty acid content in the *cti* mutants could be observed under short-day conditions. In contrast, increased fatty acid content was measured in *cti* leaves under long-day conditions and under constant light (Fig. 5c). Like the ^14^C-acetate labeling assay, fatty acid quantification suggested that CTIs inhibit FAS in the light. We finally tested whether a light-regulated CTI/α-CT interaction was responsible for the modulation of FAS. A split-luciferase assay was implemented in leaves of *N. benthamiana*. In leaves of plants grown 4 d under continuous light after agroinfiltration, a strong split-luciferase (LUC) signal could be detected (Fig. 5d). When agroinfiltrated leaves were kept 2 d in the light then 2 d in the dark, interactions between α-CT and the CTIs appeared significantly weaker. However, a further 24 h recovery period in the light was sufficient to restore equivalent levels of α-CT/CTI interactions in all the leaves, regardless of the light/dark regime previously imposed (Fig. 5d, e and Supplementary Fig 14). Light-dependent variations in CTI1/α-CT interactions detected in split-luciferase assays were not due to differential protein accumulation (Supplementary Fig. 14a). To further demonstrate the light-dependence of CTI1/α-CT interaction, co-immunoprecipitation experiments were carried out using leaf samples harvested after different light/dark treatments. In agreement with split-luciferase assays, an extended (24 h) dark period reduced the interaction between α-CT and CTI (Fig. 5f). A strong interaction between the proteins could be restored by an extended 16 h light illumination. These observations suggest that light-dependent CTIs/α-CT interactions play an important role in the regulation of htACCase and FAS. In summary, we identified a membrane regulatory subunit to the plant htACCase, and light appears to trigger both positive^5,6^ and negative regulatory mechanisms targeting FAS. This is evident from light-induced CTI/α-CT interactions attenuating htACCase activity and limiting carbon flux into de novo FAS (Fig. 5g). We suggest this permits the plant cell to finely regulate the allocation of products of photosynthesis, thus optimizing carbon and energy homeostasis in response to changing environmental and developmental parameters.

## Discussion

De novo fatty acid synthesis in higher plants occurs mainly in plastids, and is catalyzed by a series of enzymes and acyl-carrier proteins^33^. For thermodynamic efficiency, it was proposed that the enzymes, substrates and cofactors for FAS may be compartmentalized to form a metabolic channel^36^. However, we lack a clear picture of such a FAS metabolon. Another dilemma regards how does de novo FAS in the plastids ‘communicate’ with the cytosol to meet the acyl chain demand of the entire cell? One possibility would be that FAS enzymes sense cellular demand across the envelope by directly associating with this membrane conduit^20^. The carboxylation of acetyl-CoA to malonyl-CoA by ACCase is the first committed step of FAS, and is considered rate-limiting for FAS^37^. ACCase was detectable on the inner envelope membrane of pea plastids^38^. Subsequently, a protein of 96 kDa (IEP96) associated with the chloroplast inner envelope membrane was characterized^18,19^. IEP96, which shares high similarity with the 35 kDa α-carboxyltransferase subunit of ACCase from *Escherichia coli*, proved to be the α-CT subunit of plant htACCase^18,19^. The CT subcomplex of htACCase is tightly membrane associated whereas BC/BCCP subcomplex can be partially dissociated from chloroplast membranes^20^. Because of the strong association of α-CT with the chloroplast envelope^19,20^ and the lack of a predicted transmembrane domain in α-CT, it was proposed that a chloroplast membrane protein may mediate such membrane association of α-CT^20^. In this study, we used the noncatalytic domain of α-CT as bait in a yeast two-hybrid screen, and we identified an uncharacterized gene, that we termed *CTI1*. A survey of the Arabidopsis genome revealed two genes related to *CTI1*, *CTI2*, and *CTI3* (Supplementary Fig.2). All three CTI proteins localize to the plastid envelope membrane and interact specifically with the noncatalytic domain of α-CT (Figs. 1, 2). The thorough characterization of *cti* mutants generated by CRISPR/Cas9 technology showed that CTI1 is the predominant isoform with respect to the molecular function of the CTIs (Fig. 3). This could be attributed to (i) a higher expression level of *CTI1* (Supplementary Fig. 11) combined with (ii) a higher affinity of CTI1 to α-CT (Fig. 1c). In agreement with these observations, CTI1 appears to have coevolved with htACCase. The occurrence of CTI2/CTI3 in graminaceous monocots (Supplementary Fig. 2), which lack htACCase, then raises the unresolved question of the function of these homologs in such species.

While FAS in leaves is essential to support the production of membrane lipids and waxes, overproduction of FAs can lead to membrane damage so that excess FAs are detoxified via TAG biosynthesis and accumulation, or via degradation through beta-oxidation pathway^39^. TAGs function as a buffer for cytotoxic FAs and other lipid intermediates, thereby playing a key role to maintain intracellular lipid homeostasis^39,40^. Leaf TAG production is energetically wasteful though^39,40^. Under normal circumstances, FAS is highly regulated in vegetative tissues to minimize TAG production^40^. The α-CT/CTI module clearly participates in this regulatory mechanism: illumination promotes α-CT/CTI interactions, which in-turn attenuates htACCase activity, thus reducing carbon flux into FAS (Fig. 5). Decreased C18 fatty acid desaturation ratio was observed in both in leaves and seeds of *cti* mutants. This phenotype probably originates from the disconnection between the FAS module in the plastid and FA modification/elongation modules in the endoplasmic reticulum, the location of production of polyunsaturated fatty acids (PUFAs) and very long chain fatty acids (VLCFAs). FA modifications seem to function at the same rate regardless of de novo FA production capacity. In seeds of Arabidopsis *pII* mutants^16^ and transgenic rapeseed lines overexpressing *ACC1* in plastids^41^, increased ACCase activity was associated with decreased proportion of polyunsaturated FAs, just like in *cti* mutants. Reciprocally, in *wri1* mutant seeds, FA production is decreased, and the relative proportions of PUFAs and VLCFAs are higher^16,29^.

Light exposure upregulates FAS by activating htACCase^4,5,37^. Illumination first creates optimal physiological conditions for htACCase by raising pH and concentrations of Mg^2+^, ATP, and reductants. Light-regulated post-translational modifications of catalytic subunits of htACCase have also been described^42^. More recently, pH modulation of BADC/BCCP association within the biotin carboxylase subcomplex was identified and proposed to contribute to light-dependent regulation of the activity of htACCase^11^. BADC subunits were shown to inhibit ACCase in the dark through dark-favored interactions with BC, which correlates with lower htACCase activity and FAS in the dark^11^. To study the effects of light on CTI-mediated FAS regulation, we carried out ^14^C-acetate labeling assays to quantify the FAS rates. ^14^C-acetate labeling in FAS is light-dependent, and has been proposed to be achieved by light-dependent carboxylation of acetyl-CoA by htACCase^31–33^. Like other studies, we found that ^14^C-acetate labeling is a very practical approach to study htACCase in vivo^6,7,33,43^. It revealed that CTIs attenuate the rate of FAS in the light. In addition, ^14^C-acetate labeling suggests that both the accumulation of fatty acids in leaves of *cti* mutants and CTI/α-CT interactions are regulated by light (Fig. 5). Altogether, these data demonstrate that interactions of CTI isoforms with the α-CT subunits regulate FAS in a light-dependent manner.

Phosphorylation of α-CT was previously described and two phosphorylation sites (Ser741 and Ser744 on Arabidopsis ortholog) were identified by phosphoproteomic studies^44,45^. Both of the identified α-CT phosphorylation sites are near the C-terminus, within the noncatalytic domain of the protein, which is responsible for its interaction with CTI. Additionally, a different phosphoproteomic study identified phosphorylation sites on the the C-terminus of CTI1 (Ser160, Thr161) and CTI3 (Thr151, Ser152, and Ser153)^46^. The identified phosphorylation sites are conserved in all three CTIs. Protein phosphorylation can be used to modulate the nature and the strength of protein-protein interactions thereby regulating protein binding^47^. Considering the occurrence of phosphorylation sites in the protein binding regions (coiled-coil domains) of both α-CT and CTIs, it would be interesting to further study whether such protein modifications affect the dynamics of CTI/α-CT binding.

Vegetative tissues in *cti1* mutants display measurable TAG accumulation, resulting in at least a four-fold increase in extractable leaf oil compared with wild-type *Arabidopsis* leaves (Fig. 3h). From a biotechnological point of view, CTIs may thus represent promising targets for the engineering of vegetative oil crops. While oilseed-based fuel production can meet only a small proportion of world transportation needs before impinging on the global food supply, the overproduction of oil in non-seed tissues such as leaves and stems may be exploited as part of an overall strategy to maximize the recovery of renewable resources from agricultural products^39,48^.

## Methods

### Plant material and growth conditions

*Arabidopsis thaliana* accession Col-0 was used as wild-type control in this study. The T-DNA mutant line (*cti1-2*; N599125) was ordered from ABRC (Arabidopsis Biological Resource Center). Characterization of the *wri1-3* and *wri1-4* T-DNA insertion lines and construction of the *Pro35Sdual:WRI1* lines (T63 and T81) were previously reported^28^. All plants were grown in growth chamber at 22 °C. Light intensity was 82–115 (98 on average) μmol m^−2^ s^−1^ and humidity was 50%. The long-day (LD) photoperiod corresponded to an alternate 16 h light/8 h dark regime. The short-day (SD) photoperiod corresponded to an alternate 12 h light/12 h dark regime.

### Constructs preparation and stable transformation of Arabidopsis

The primers used for construct preparation are listed in Supplementary Table 3.

We designed single-guide RNAs (sgRNA) for CRISPR/Cas9 using the online CRISPR-P 2.0 software^49^. We annealed the oligo pairs to generate double-stranded DNA and cloned the sgRNAs into the *Bbs*I site of the gateway compatible entry vector U6-sgRNA. We then introduced the U6-sgRNA cassette into a modified pCambia1300 binary vector, in which *hspCas9* expression is driven by the *YAO* promoter^50^.

To construct *ProCTI1:CTI1:uidA*, *ProCTI2:CTI2:uidA*, and *ProCTI3:CTI3:uidA* transgenes, gene fragments extending from up to 2 kb upstream of the ATG of the gene to the last coding codon were amplified with the Pfu Ultra DNA polymerase (Stratagene) from Col-0 genomic DNA. The PCR products were introduced by BP recombination into the pDONR207 entry vector (Invitrogen) and transferred into the destination vector pBI101-R1R2-GUS^51^ by LR recombination. The resulting binary vector was electroporated into *Agrobacterium tumefaciens* C58C strain and used for transformation^51^. Depending on the construct considered, between 17 and 19 independent transgenic lines were analyzed. To construct the *ProCTI1:uidA* transgene, region -994 to -1 bp relative to the *CTI1* translational start codon was introduced by BP recombination into the pDONR207 entry vector as described above, and transferred into the destination vector pBI101-R1R2-GUS by LR recombination. Construction of the *ProBCCP2:uidA* transgene^28^, just like that of the *ProODD:uidA* transgene^52^ followed a similar procedure.

To construct *ProAT2S2:CTI* transgenes, *CTI* cDNAs were amplified from a mixture of seed cDNA (Col-0 accession), cloned into the pDONR207 as described above, and finally transferred to the binary vector ProAT2S2-R1R2-HYGRO^53^ by LR recombination. The corresponding binary vectors were used for agroinfiltration of *cti1-2* flower buds. T2 seeds were subjected to segregation analyses for hygromycin resistance and lines segregating 3:1 were selected (heterozygous lines, one insertion locus). T2 lines were then grown in a greenhouse and their progeny (T3 seeds) was subjected to segregation analyses. Lines producing 100% resistant plantlets were selected (homozygous lines, single insertion locus) and used for further characterization.

To construct *Pro35S:CTI1:YFP*, *Pro35S:CTI2:YFP*, *Pro35S:CTI3:YFP*, and *Pro35S:*α*-CT:YFP* transgenes for protoplast transient transformation, *CTI* and α*-CT* coding sequences (CDSs) without STOP codons were amplified from a mixture of cDNA (Col-0 accession), cloned into the pENTR/D-TOPO, and transferred into the pAM-PAT-35SS::YFP:GW vector^54^ by LR recombination.

To construct *Pro35S:CTI1:GFP*, *Pro35S:*α*-CT:RFP*, and *Pro35S:TGD2:RFP* transgenes, CDSs were cloned into the pENTR/D-TOPO vector as above, before being transferred into destination vectors pGWB605 (GFP) and pGWB654 (RFP) by LR recombination^55^.

To construct BiFC and split-luciferase vectors, CDSs previously cloned into the pENTR/D-TOPO were transferred into pAM-PAT-35SS::cYFP/nYFP:GW, and pEarley-nLUC/cLUC vectors by LR recombination.

To purify the coiled-coil domains of the CTIs and to perform yeast two-hybrid assays, the CDSs corresponding to CTI coiled-coil domains were amplified by PCR, cloned into the pENTR/D-TOPO entry vector and transferred into destination vectors pGADT7-GW by LR recombination.

To purify the proteins for microscale thermophoresis, the C-terminus of CTI1, CTI3, and α-CT were each fused with a HIS tag, and cloned into pET30a (CTI3 and α-CT) or pDEST17 (CTI1). CTI2 was fused with a GST tag and cloned into the pGEX4T-1 vector.

To construct the *Pro35S:CTI1:HA* transgene, a *Hind*III/*Stu*I fragment of the pGWB14 binary vector^55^ containing a 35S promoter-R1-CmR-ccdB-R2-3xHA-NosT sequence was *Hind*III/*Sma*I inserted in the pBluescript KS(+) vector (Stratagene), giving the pBKS-35S-R1R2-3xHA vector. *CTI1* CDS without STOP codon previously cloned into the pDONR207 (see above) was transferred into the pBKS-35S-R1R2-3xHA vector by LR recombination. To construct the *ProCTI1:CTI1:HA* transgene, the *CTI1* gene was amplified from Col-0 genomic DNA and the 3xHA tag was amplified from the pBKS-35S-R1R2-3xHA containing the *Pro35S:CTI1:HA* fusion (see above). The two amplified fragments were combined together to serve as overlapping templates for a PCR reaction utilizing primers listed in Supplementary Table 3. The final PCR product was cloned in pDONR207, and finally transferred into the binary vector pBIB-Hyg-GTW^51^ by LR recombination. The corresponding binary vector was used for agroinfiltration of *cti1-2*. Homozygous lines (single insertion locus) were selected as described above.

To construct the *Pro35S:TOC33:MYC* transgene, the CDS corresponding to TOC33 was amplified by PCR, cloned into the pENTR/D-TOPO entry vector and transferred into destination vectors pGWB617 by LR recombination.

To construct the *Pro35Sdual:WRI1*^51^ and *Pro35Sdual:MYB118* transgenes^52^, the corresponding CDSs were amplified by PCR, cloned into the pDONR207 entry vector by BP recombination and transferred into the pMDC32 destination by LR recombination.

### RNA isolation and quantitative real-time PCR

For RNA extraction, frozen tissues were ground in liquid nitrogen and total RNA was extracted using the RNeasy Plant Mini Kit (Qiagen) according to the manufacturer’s instructions. For reverse transcription, RNA was converted into first-strand cDNA using the SuperScript preamplification system for first-strand cDNA synthesis (ThermoFisher) with oligo(dT)_22_. The real-time quantitative RT-PCR reaction was performed on the LightCycler Instrument (Roche) with the LightCycler-FastStar DNA Master SYBR Green I kit for PCR (Roche) according to the manufacturer’s protocol. Each reaction was performed with 5 µL of 1:50 (v/v) dilution of the first cDNA strands in a total volume of 20 µL. The reaction was incubated at 95 °C for 8 min to activate the hot start recombinant *Taq* DNA polymerase, followed by 45 cycles of 10 s at 95 °C, 6 s at 55 °C, and 20 s at 72 °C^56^. Specific primer sequences are presented in Supplementary Tables 4, 5. The specificity of the PCR amplification was checked with a heat dissociation protocol (from 65 to 95 °C) following the last cycle of the PCR.

### Yeast one-hybrid assay

Construction of the *ProCTI1-500:HIS* reporter plasmid, like that of the *ProBCCP2-180:HIS* reporter plasmid^29^, relied on the amplification by PCR of promoter fragments, that were digested by *Eco*RI and *Sac*II, and inserted into the pHISi vector between the *Eco*RI and *Sac*II sites. The plasmids thus obtained were digested with *Nco*I and integrated into the yeast strain YM4271 at the *URA3* locus. Yeast cells presenting the *HIS3* reporter gene under the control of the different promoters studied were transformed with pDEST22 using LiAc/SSDNA/PEG method. Yeast cells were grown in YPDA medium and harvested by centrifugation at 3000 × *g*. For each transformation, around 10^8^ cells were mixed with T Mix (240 μL PEG 3350 50% (w/v), 36 μL 1 M lithium acetate, 50 μL boiled SS-Carrier DNA (2 mg/mL), 34 μL plasmid DNA (0.1–1 μg)). After vigorous mix by vortex, the mixtures were incubated in a 28 °C water bath for 30 min, then in a 42 °C water bath for 25 min. Afterwards, cells were pelleted by centrifugation, washed with sterile water, resuspended in 200 µL sterile water before being plated on selective medium and grown at 30 °C for 3 d.

### Yeast two-hybrid assays

The cDNA library used was prepared by introducing Arabidopsis cDNA prepared from seedlings into the pGATD7-GW vector. The screening assay was conducted according to the Clontech yeast handbook. Briefly, the sequence encoding the coiled-coil domain of α-CT (amino acid 420–769) was cloned into the pGBKT7 vector. This vector was used to transform the AH109 yeast strain. Yeast transformants were selected on synthetic dropout (SD) medium lacking tryptophan. Then, 100 μg of cDNA library were used to transform AH109 cells containing the pGBKT7 vector expressing the coiled-coil domain of α-CT. Cells plated on SD medium lacking leucine, tryptophan, histidine, adenine, were incubated at 30 °C for 4 d and cDNAs of positive clones were amplified by PCR and sequenced using T7 and 3’AD primers (Supplementary Table 6).

For oriented interaction assays, the sequences coding for the coiled-coil domain of CTIs were cloned into the pGADT7 vector as described above. Then, the AH109 strain was cotransformed with the pGADT7 vector expressing CTI coiled-coil domains and the pGBKT7 vector expressing the α-CT coiled-coil domain, before being plated on SD medium lacking leucine and tryptophan. After 3 days at 30 °C, positive clones were transferred to SD medium lacking leucine, histidine, and tryptophan, in the presence of 1 mM 3AT for another 4 d at 30 °C.

### Transformation of Arabidopsis protoplasts

Arabidopsis protoplasts were prepared from 4-week-old Col-0 plants^57^. About 40 leaves were cut into strips with a sharp razor blade and then transferred into 20 mL enzyme solution (20 mM MES pH 5.7, 1.5% (w/v) cellulase R10, 0.4% (w/v) macerozyme R10, 0.4 M mannitol, 20 mM KCl and 0.1% BSA) for 3 h at room temperature. After 3 h, the enzyme solution was filtered through 75-μm nylon mesh that was then washed with 20 mL W5 solution (2 mM MES pH 5.7, 154 mM NaCl, 125 mM CaCl_2_, 5 mM KCl). The flow-through was centrifuged at 200 × *g* for 2 min. After removing as much supernatant as possible, the protoplast pellet was resuspended in 1 mL W5 solution and kept on ice for 30 min. The protoplasts were pelleted at 200 × *g* for 2 min and resuspended in 500 μL MMG solution (4 mM MES pH 5.7, 0.4 M mannitol, 15 mM MgCl_2_). Then, 10 μL purified plasmid (10-20 μg) were mixed with 100 μL protoplasts before 110 μL PEG solution, namely 40% (w/v) PEG4000 in ddH_2_O containing 0.2 M mannitol and 100 mM CaCl_2_, were added. The transfection reaction was mixed by gently tapping the tube and incubated at room temperature for 10 min. The mixture was then diluted in 500 μL W5 solution, mixed by gently rocking the tube to stop the transfection process, and ultimately centrifuged for 2 min at 200 × *g*. The pellet was resuspended in 1 mL W5 solution and incubated overnight at 23 °C under weak light.

### Transient expression in leaves of *Nicotiana benthamiana*

*Agrobacterium tumefaciens* strains transformed with the different binary vectors prepared were grown overnight in selective medium. Cells were then pelleted, washed two times and resuspended in injection buffer (50 mM MES pH 5.7, 10 mM MgCl_2_). Different strain combinations were coinfiltrated into the leaves of *Nicotiana benthamiana* in the presence of a vector coding for the P19 protein of tomato bushy stunt virus (TBSV) that prevents the onset of post-transcriptional gene silencing.

### Confocal microscopy

Fluorescence was observed by confocal laser microscopy using a Leica TCS SP8 device. Fluorescence were observed at 488–505 nm for GFP, 514–527 nm for YFP, and 558–583 nm for RFP.

### Split-luciferase assay

After agrobacterium-mediated infiltration of *N. benthamiana* leaves, different light/dark treatments were applied and luciferase activity was detected with a CCD camera by applying firefly D-luciferin (Goldbio). For 24-h light recovery assays, after luminescence imaging, the detached leaves were kept in a tray with water to maintain humidity. After 24 h of light recovery, the luciferase activity was detected with the CCD camera again. Ponceau S Staining (1% (w/v) Ponceau S, 20% acetic acid) was used to quantify relative protein contents.

### Microscale thermophoresis (MST)

The coiled-coil domains of CTI1, CTI3, and α-CT fused with HIS tag were purified by capture on nickel agarose resin. The coiled-coil domain of CTI2 fused with a GST tag was purified by capture with glutathione resin. Each protein was expressed in Luria Broth. Expression was induced with 100 μM IPTG when OD_600_ reached 0.6. The cells were pelleted and resuspended with lysis buffer (PBS buffer (137 mM NaCl, 2.7 mM KCl, 10 mM Na_2_HPO_4_ and 2 mM KH_2_PO_4_, pH 7.4). After cell disruption by French press and centrifugation, the proteins were purified by nickel agarose resin (Gold Biotechnology, H-320) or glutathione agarose resin (Gold Biotechnology, G-250). The purified protein samples were concentrated to 500 μL with a Pierce concentrator PES with a 3000 Dalton cutoff (ThermoFisher, 88527). The GST tag was proteolytically separated from CTI2 using thrombin treatment. Proteins were quantified by Bradford assays using bovine gamma globulin as a standard. 100 nM α-CT-His protein was fluorescently labeled using His-tag labeling kit (NanoTemper, MO-L008) according to the user manual. After labeling, 10 μL of labeled α-CT protein was mixed with 10 μL of CTI (serial dilutions in PBS buffer). The microthermophoresis was carried out using 100% LED power and high MST power with a NanoTemper monolith NT.115 instrument. The data were analyzed by MO.Offinity Analysis (X86).

### Immunoblotting

For quantification of the htACCase subunit content, proteins were extracted from crude chloroplasts using a protein extraction buffer (50 mM HEPES-KOH pH 7.6, 150 mM NaCl, 1 mM EDTA, 10% (v/v) glycerol, 1 mM 2-mercaptoethanol, 1% (v/v) Triton X-100, 1X complete protease inhibitor cocktail). The crude chloroplasts were isolated from 5 g of 4-week-old Col-0 leaf tissues. The proteins were separated by SDS-PAGE and blotted to a PVDF membrane. The BCCP, α-CT and β-CT subunits of plastidic htACCase were immunologically detected with dedicated antisera^19^. A horseradish peroxidase-conjugated secondary antibody was used and the horseradish peroxidase activity was detected by enhanced chemiluminescence (ECL) western blotting substrate (ThermoFisher; Cat. No. 32106). Ponceau S Staining was used as a loading control.

### Co-immunoprecipitation

Crude chloroplasts were isolated from 4-week-old Col-0 plants. Around 5 g of fresh leaves were ground in 30 mL ice-cold isolation buffer (50 mM HEPES pH 8.0, 2 mM EDTA, 2.5 mM MgCl_2_, 5 mM NaHCO_3_, 0.33 M sorbitol, 0.5 % BSA). After filtration of the homogenate through miracloth, the flow-through was centrifuged at 1000 × *g* for 10 min at 4 °C. The pellet was resuspended in 1 mL of protein extraction buffer containing 50 mM Tris pH 7.5, 150 mM NaCl, 1% Triton-X 100 and 1x protease inhibitor cocktail (Sigma). After a 30-min incubation period on ice, the solution was centrifuged at 20,000 × *g* for 15 min at 4 °C. The supernatant was decanted before addition of 5 μL of anti-α-CT antibody and subsequent incubation for 4 h with end-to-end rotation at 4 °C. After addition of 25 μL of protein A resin (Genescript; Cat. No. L00210), another 2 h incubation period was implemented. The mixture was finally spun down and washed three times with washing buffer (50 mM Tris pH 7.5, 150 mM NaCl). After washing, the resin was incubated with 100 μL of 1× SDS-PAGE loading buffer, and then heated at 80 °C for 10 min. Proteins were separated by SDS-PAGE and then transferred to PVDF membrane. After transfer, the proteins were detected by immunoblotting with anti-CTI1 or anti-α-CT antibody. A horseradish peroxidase-conjugated secondary antibody was used and the horseradish peroxidase activity was detected by ECL western blotting substrate (ThermoFisher; Cat. No. 32106).

### Protease protection assay

Four-week-old leaves of transgenic plants stably expressing a TOC33-MYC fusion were harvested and 10 g of fresh material were used to isolate crude chloroplasts according to method described above. Chloroplasts were resuspended in 1 ml of isolation buffer and loaded onto a Percoll gradient. The Percoll gradient was obtained by mixing 15 mL of Percoll with 15 mL of 2× isolation buffer, and centrifuging the mixture at 38,700 × *g* for 30 min at 4 °C. The chloroplasts were centrifuged on the Percoll gradient using a prechilled swinging bucket rotor at 7700 × *g* for 10 min at 4 °C with no brake. After centrifugation, the upper green band corresponding to broken chloroplasts was removed and discarded. The lower green band was transferred into a new 50-mL centrifuge tube containing 10 mL of isolation buffer. The mixture was spun down at 1500 × *g* for 5 min at 4 °C, and the pellet resuspended in 1 mL reaction buffer (50 mM HEPES pH 8.0, 0.33 M sorbitol). The proteolytic digestions were set up as follows: mock—150 μL of chloroplasts were mixed with 100 μL of reaction buffer; thermolysin—150 μL of chloroplasts were mixed with 10 μL of thermolysin stock solution (1 mg/mL, freshly prepared in 5 mM CaCl_2_ in reaction buffer) and 90 μL of reaction buffer; trypsin—150 μL of chloroplasts were mixed with 10 μL of trypsin stock solution (1 mg/mL, freshly made in reaction buffer) and 90 μL of reaction buffer. All reactions were incubated on ice for 30 min. Each proteolytic reaction was quenched on ice for 5 min as follows: mock—50 μL of reaction buffer was added; thermolysin—50 μL of quench solution (60 mM EDTA in reaction buffer) was added; trypsin—50 μL of trypsin inhibitor solution (1 mg/mL in reaction buffer; Sigma; Cat. No. T6522) was added. SDS-PAGE loading buffer was then added to each reaction. Western blotting was performed as described above using anti-Myc, anti-α-CT or anti-CTI1 antibodies.

### Immunolocalization experiments

Coverslips were sterilized in 95% ethanol and dried before coating. They were then incubated for 5 min at 37 °C in a 50 µg/mL poly-L-lysine solution before drying. Arabidopsis protoplasts were fixed for 30 min with 3% (w/v) paraformaldehyde in PBS supplemented with 30.5 g/L glucose and 30.5 g/L mannitol (pH 7.4; PBS-GM) at room temperature. Fixed protoplasts were washed three times with 50 mM ammonium chloride in PBS-GM. The final suspension was plated onto poly-L-lysine-coated coverslips. Protoplasts were allowed to settle for 10 min at room temperature, and were then air dried for 1 h under a culture hood. To permeabilize protoplasts, a 15 min treatment with 0.5% (v/v) Triton X-100 buffered with PBS-GM was carried out. After three washes with PBS-GM, nonspecific binding was blocked with 1% bovine serum albumin in PBS-GM for 1 h. Protoplasts were then incubated overnight at 4 °C with rabbit anti-E37 antibodies^19^ (1:500) followed by 1 h incubation at room temperature with rat anti-HA antibodies (1:2000; Roche). Cells were washed for 20 min in PBS-GM, and incubated with goat anti-rabbit immunoglobulin G-Alexa Fluor 568 (1:1000; Molecular Probes) and with goat anti-rat immunoglobulin G-Alexa Fluor 488 (1:500; Molecular Probes) or with goat anti-mouse immunoglobulin G-Alexa Fluor 488 (1:500; Molecular Probes) for 1 h. Finally, cells were washed for 20 min in PBS and mounted using Vestashield mounting solution (Vector Laboratories). Cells were visualized with an inverted spectral confocal laser microscope (LEICA SP2-AOBS). Preparation of embryos excised from maturing seeds was similar to that of protoplasts except for the permeabilization treatment: cell walls were partially digested by incubating embryos in 2% (w/v) driselase (Sigma) in PBS-GM for 45 min, then membranes were permeabilized by a 1 h treatment with 10% (v/v) DMSO and 3% (v/v) NP-40 in PBS-GM.

### Lipid and fatty acid analyses

To analyze polar lipids and triacylglycerols, 500 mg of leaves were harvested and stored at −80 °C prior to lipid extraction. Leaf samples were ground in 7.2 mL of precooled chloroform/methanol/formic acid (10:10:0.5, v/v/v) and incubated at -20 °C overnight. The mixture was centrifuged at 7500 × *g* for 10 min at 4 °C to pellet the cell debris. Lipids of the pellet were reextracted in 2.64 ml of precooled chloroform/methanol/water (10:10:1, v/v/v). Then, 3.6 mL of cooled Hajra solution (2 M KCl and 0.2 M H_3_PO_4_) were added to the pooled supernatants. After shaking and centrifugation (7500 × *g* for 10 min at 4 °C), the lower phase containing lipids was collected and evaporated with a stream of N_2_. Lipids were resuspended in chloroform/methanol (2:1, v/v) and separated on thin-layer chromatography plates developed with hexane/diethylether/acetic acid (35:15:0.01, v/v/v). Polar lipids and triacylglycerols were visualized under UV light by staining with sprayed primuline. Lipid spots were ultimately collected and analyzed by gas chromatography^58^.

To analyze total fatty acids in leaves or seeds, total lipids were transmethylated into fatty acid methyl esters (FAMEs). FAMEs were analyzed by a Hewlett Packard 6890 gas chromatograph. Leaves from 4-week-old plants were collected and dried by centrivap SpeedVac overnight. Mature seeds were harvested and dried at room temperature for 1 week prior to analysis.

### Lipidomics

Lipid extracts were dissolved in chloroform, and appropriate amounts of internal standards were introduced. The lipids were detected by triple quadrupole mass spectrometer (Applied Biosystems API 4000) with an autosampler (LC Mini PAL; CTC Analytics)^59^. Data processing was performed in a Lipidome DB Data Calculation Environment (http://lipidome.bcf.ku.edu:9000/Lipidomics) with normalization to internal standards.

### In vivo acetate labeling

In vivo labeling experiments were performed according to previous report^60^. Leaves from 4-week-old plants were cut into strips after measuring the fresh weight. The leaf strips were transferred into 2 mL of reaction buffer (20 mM MES pH 5.5, half MS, 0.01% (v/v) Tween 20) in a 6-well plate. The labeling assays were started by the addition of 1 μCi of ^14^C-acetate (PerkinElmer; Cat. No. NEC084H001MC) or 1 μCi of ^14^C-acetate (Morevek; Cat. No. MC213). After this, the 6-well plates were incubated on a shaker in the light (40 µmol m^−2^ s^−1^) at room temperature or in the dark. The samples were collected after 40 min of incubation, washed three times with water and total lipids were then extracted in 800 µL of methanol/chloroform/formic acid (20:10:1, v/v/v) with vortexing for 10 seconds^61^. The mixture was incubated for 30 min at 22 °C, complemented with 500 µL of 1 M KCl and 0.2 M H_3_PO_4_, vortexed and centrifuged at 12,000 × *g* for 30 sec for collection of the chloroform phase containing the lipids. One milliliter of scintillation cocktail was added to this phase before measurement of the incorporated radioactivity (in cpm) with a scintillation counter.

### ACCase activity assay

ACCase activity was directly quantified from 4-week-old leaves by incorporation of H^14^CO_3_ into acid-stable products. Around 5 g of leaf tissues were harvested. Crude chloroplasts were isolated as above, and chloroplast proteins were extracted using 300 μL of extraction buffer (50 mM Tris-HCl pH 7.5, 100 mM KCl, 5 mM MgCl_2_, 1 mM DTT, 1% (v/v) Triton X-100, 10% (v/v) glycerol, and 1× plant protease inhibitor mixture from Sigma). After centrifugation at 13,000 × *g* for 10 min, the supernatants were desalted with spin desalting columns (ThermoFisher; Cat. No. 89882). The insoluble pellets were resuspended in the extraction buffer. Reactions were initiated by mixing 10 μL of reaction buffer (100 mM Tricine pH 8.2, 100 mM KCl, 15 mM ATP, 5 mM MgCl_2_, 1 mM DTT, 2.5 mM acetyl-CoA, 50 μM haloxyfop, and 1 μCi ^14^C-NaHCO_3_ (Moravek; Cat. No. MC208) with 40 μL of protein extract. After 30 min at RT, the reactions were quenched by adding 50 μL of 12 N HCl. The reaction solutions were transferred into scintillation vials with a filter paper at the bottom of the vial. Samples were dried at 80 °C for 1 h, after which 1 mL of scintillation cocktail was added and radioactivity counted by liquid scintillation. Controls without acetyl-CoA were included to determine and subtract nonspecific background rates.

### GUS assay

For histochemical detection of GUS activity, tissues were incubated in 0.1 M phosphate buffer, pH 7.2 containing 2 mM 5-bromo-3-indolyl-β-D-glucuronide, 0.1% (v/v) Triton X-100, 10 mM Na_2_-EDTA, and 0.2 (Arabidopsis vegetative organs, flowers, and seeds) or 2 mM (Arabidopsis embryos and *N. benthamiana* leaf disks) each of potassium ferricyanide and potassium ferrocyanide. A vacuum was applied for 1 h before incubating the samples for 4 h (Arabidopsis embryos and *N. benthamiana* leaf disks) or 15 h (Arabidopsis vegetative organs, flowers, and seeds) at 37 °C in the dark, and chlorophyll was finally removed by room temperature incubation in 70% (v/v) ethanol (vegetative tissues and flowers only).

### Reporting summary

Further information on research design is available in the Nature Research Reporting Summary linked to this article.

## Supplementary information

Supplementary Information

Reporting Summary

## Data Availability

The authors declare that the data supporting the findings of this study are available within the article and its Supplementary Information files. The datasets and plant materials generated and analyzed during the current study are available from the corresponding author upon reasonable request. Sequence data from this article can be found in TAIR under accession numbers: *AT2S2*, At4g27150; *BAN*, At1g61720: *BC*, At5g35360; *BCCP1*, At5g16390; *BCCP2*, At5g15530; *CTI1*, At1g42960; *CTI2*, At3g02900; *CTI3*, At5g16660; *α-CT*, At2g38040; *β-CT*, AtCg00500; *EF1αA4*, At5g60390; *MYB118*, At3g27785; *ODD*, At1g04380; *PTST2*, At1g27070; *TGD2*, At3g20320; *TOC33*, At1g02280; *WRI1*, At3g54320; *WRI2*, At2g41710. Source data are provided with this paper.

## Source data

Source Data
