# Supplementary material for: Docking of acetyl-CoA carboxylase to the plastid envelope membrane attenuates fatty acid production in plants
Source: Nat Commun. 2020 Dec 3;11:6191. doi: 10.1038/s41467-020-20014-5 (PMC7712654; doi:10.1038/s41467-020-20014-5)
Supplement: Supplementary file 1 — Supplementary Information [file 41467_2020_20014_MOESM1_ESM.pdf]

Supplementary Figure 1

a

	CTI1	CTI2
**CTI2**	35.71%	
**CTI3**	35.19%	65.19%

b

```
                     Transit Peptides
CTI1      MASLSSTSLSLPKNSH---QL--HPSSGFSLNP--NARCVSVSFGLNHSNKLHISAPRTK
CTI2      MCSSSSCMLLFS------DLLFVLMYNGVLAHRNFNAIRKSSTLT------VQ-TKSNRS
CTI3      ---MASCIATAPLSLSGVSQSHYVKANGLSTTTKLSSICKTSDLT------IH-KKSNRT
             :*                   .*.     .:   :  :       :: .  . .
                     Transmembrane Domain
CTI1      RILTIQSAYRDDDGSGSTGLFVGGFILGGLIVGALGCVYAPQISKAIAGADRKDLMRKLP
CTI2      HKLSVSAGYRGGSKGGGSSDFVTGFLLGSAVFGTLAYIFAPQIRRSVLS-ENEYGFKKPE
CTI3      RKFSVSAGYRDGSRSGSSGDFIAGFLLGGAVFGAVAYIFAPQIRRSVLNEEDEYGFEKPK
          : :::.:.**...  .*.:.  *: **:**.  :.*::. ::**** :::  . : :  :.*
                     Coiled-coil Domain
CTI1      KFIYDEEKALEKTRKVLAEKIAQLNSAIDDVSSQLKSEDTPNGAALST--------DEIE
CTI2      QPM-YYDEGLEERREILNEKIGQLNSAIDKVSSRLKGGRSGSSKNTSSPSVPVETDAEAE
CTI3      QPT-YYDEGLEKTRETLNEKIGQLNSAIDNVSSRLRGRE----KNTSSLNVPVETDPEVE
          :     ::.**: *: * ***.*******.***:*:.          *:          * *

CTI1      ATA
CTI2      ATA
CTI3      ATT
          **:
```

c

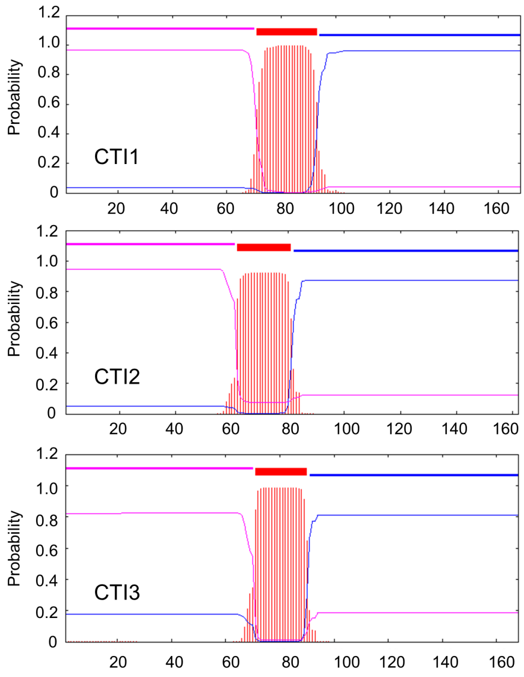

d

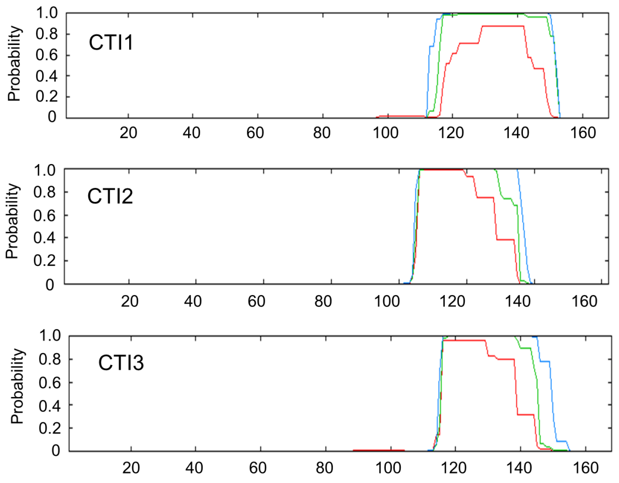

Supplementary Figure 1. Protein sequence alignments and functional domain predictions. a The three CTI protein sequences were aligned and percent amino acid identity was calculated using ClustlW. **b** Alignment of the three CTIs. Transit peptides were predicted using ChloroP, transmembrane and coiled-coil domains were predicted using the ExPASy bioinformatics resource portal. Conserved and identical residues are denoted by : and * symbols, respectively. The underlined sequence indicates the protein sequence used for yeast-two-hybrid assays. **c** The distribution of transmembrane domains was predicted by the TMHMM Server. **d** The distribution of the coiled-coil domains was predicted by an ExPASy server (Pole Bioinformatique Lyonnais).

Supplementary Figure 2

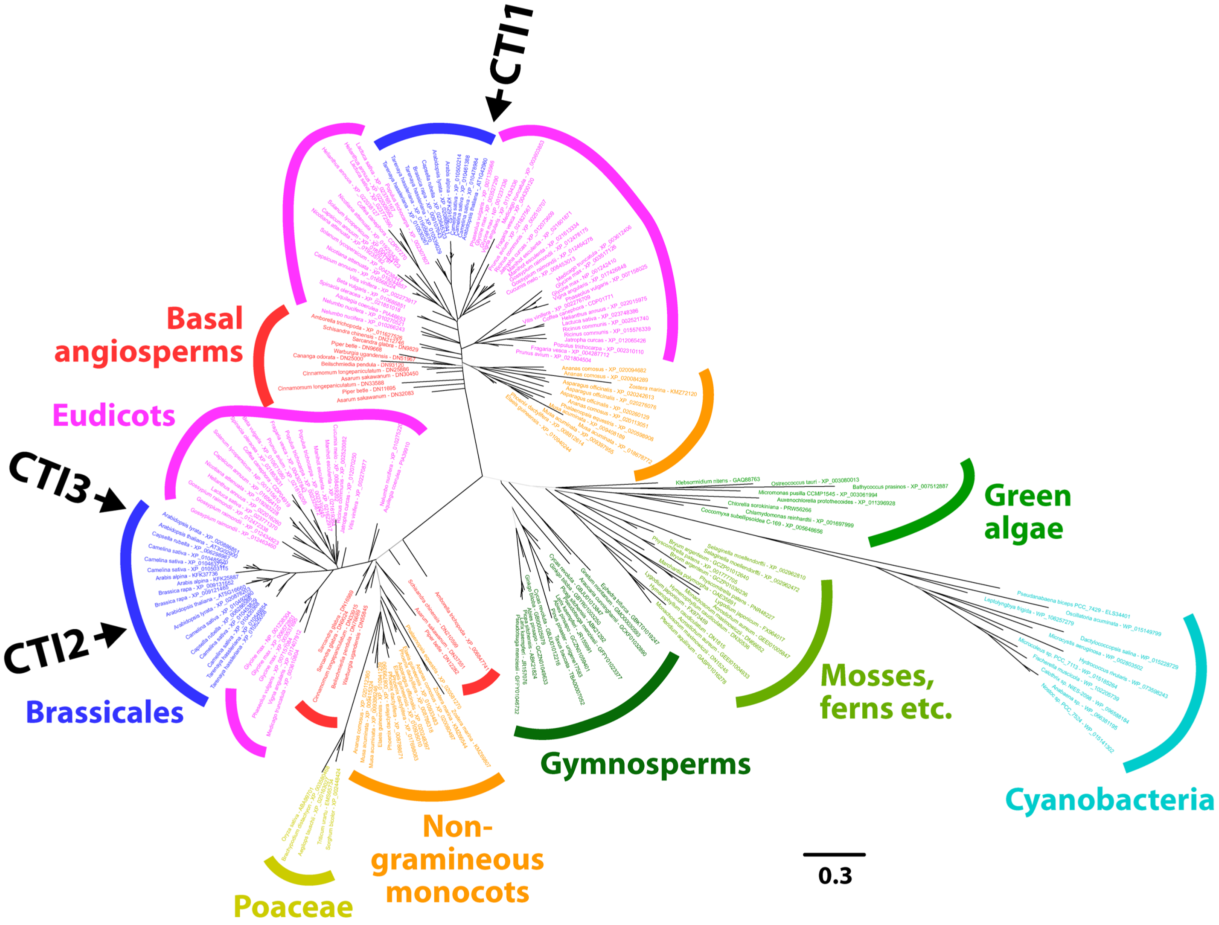

Supplementary Figure 2. Phylogenetic analysis of *CTI* genes. The phylogenetic tree includes representative non-redundant orthologs recovered via PSI-BLAST from the NCBI RefSeq database using Arabidopsis CTIs as queries, in addition to orthologous sequences retrieved via TBLASTN from the NCBI TSA database and from third-party and custom Trinity assemblies (to supplement basal angiosperm and non-angiosperm plant sequences). Open-reading frames from transcript hits were translated into amino acid sequences using TransDecoder (v. 3.0.1). Amino acid sequences were aligned with the MAAFT (v. 7.388) plugin in Geneious Prime (v. 2020.0.2) using the E-LNS-I option. Sites with 95% or more gaps were masked from the alignment, and a phylogenetic tree was constructed with RAxML (v. 8.2.10) using the Gamma protein model with RELL support values. Trees were plotted in FIGTREE (v. 1.4.4) and formatted in Adobe Illustrator. RELL support is indicated by the darkness of branch lines while clades are colored by taxonomic group. Accession numbers are displayed on the tree and in Supplementary Table 2.

Supplementary Figure 3

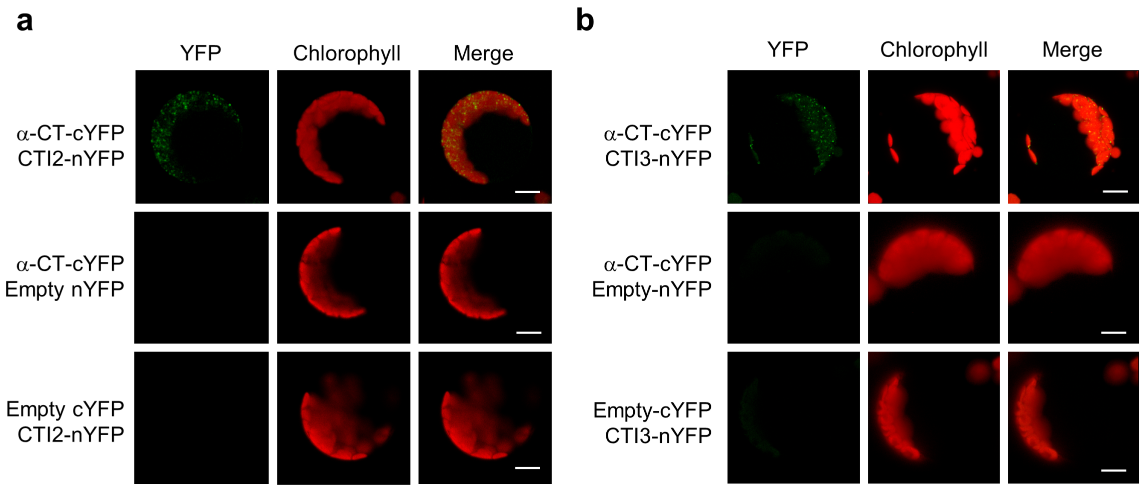

Supplementary Figure 3. CTIs interact with α-CT. a CTI2 and **b** CTI3 interact with α-CT in Bimolecular fluorescence complementation assays, in which split-YFP was used. Arabidopsis protoplasts were transformed with different construct combinations and imaged by confocal microscopy 16 h after transformation. Experiments were repeated three times with similar results. For each of the three independent protoplast transformations, 3 different protoplasts were observed. Bars = 10 µm.

Supplementary Figure 4

a

CTI1	WT	CGGTGCCCT-CGGATGTGTATATGCACCACAGGT
	cti1	CGGTGCCCTCCGGATGTGTATATGCACCACAGGT
CTI2	WT	AGCAGCCGATGTACTATGACGAAGGCCTAGAGG
	cti2	AGCAGCCGATG-ACTATGACGAAGGCCTAGAGG
CTI3	WT	CGAGTCCGGTTTGATTTCTTGTGAATAGTCAAA
	cti3	CGAGTCCGG-TTGATTTCTTGTGAATAGTCAAA

c

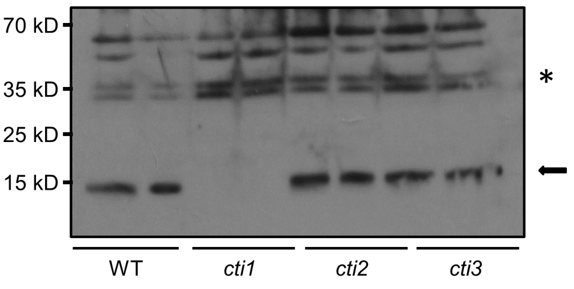

b

CTI1

WT MASLSSTSLSLPKNSHQLHPSSGFSLNPNARCVSVSFGLNHSNKLHISAPRTKRILTIQSAYRDDDGSGSTGLFVGGFIL
cti1 MASLSSTSLSLPKNSHQLHPSSGFSLNPNARCVSVSFGLNHSNKLHISAPRTKRILTIQSAYRDDDGSGSTGLFVGGFIL

WT GGLIVGALGCVYAPQISKAIAGADRKDLMRKLPKFIYDEEKALEKTRKVLAEKIAQLNSAIDDVSSQLKSEDTPNGAALS
cti1 GGLIVGALGMCICTTDQQGYSWSRPKGSHEEVA* -

WT TDEIEATA*
cti1 - - - - - - - -

CTI2

WT MCSSSSCMLLFSDLLFVLMYNGVLAHRNFNAIRKSSTLTVQTKSNRSHKLSVSAGYRGGSKGGGSSDFVTGFLLGSAVF
cti2 MCSSSSCMLLFSDLLFVLMYNGVLAHRNFNAIRKSSTLTVQTKSNRSHKLSVSAGYRGGSKGGGSSDFVTGFLLGSAVF

WT GTLAYIFAPQIRRSVLSENEYGFKKPEQPMYYDEGLEERREILNEKIGQLNSAIDKVSSRLKGGRSGSSKNTSSPSVPVET
cti2 GTLAYIFAPQIRRSVLSENEYGFKKPEQPMTMTKA* -

WT DAEAEATA*
cti2 - - - - - - - -

CTI3

WT MASCIATAPLSLSGVSQSHYVKANGLSTTTKLSSICKTSDLTIHKKSNRTRKFSVSAGYRDGSRSGSSGDFIAGFLLGGA
cti3 MASCIATAPLSLSGVSQSHYVKANGLSTTTKLSSICKTSDLTIHKKSTGLASFLFLQGIEMEVEAEAVVISLLVFF*- - - - -

WT VFGAVAYIFAPQIRRSVLNEEDEYGFEKPKQPTYYDEGLEKTRETLNEKIGQLNSAIDNVSSRLRGREKNTSSLNVPVET
cti3 -

WT DPEVEATT*
cti3 - - - - - - - -

Supplementary Figure 4. Characterization of *cti* mutants obtained using CRISP/Cas9 technology. a Molecular characterization of CRISPR/Cas9-induced *cti* mutations. Sequences targeted by the sgRNA are highlighted in red and the protospacer adjacent motif (PAM) sequences are denoted in blue. Mutations are marked in green. **b** Predicted protein sequences of CTIs in wild-type and CRISPR/Cas9 mutant backgrounds. Sequences conserved between WT and mutant proteins are highlighted in black. Red letters denote transmembrane domains and green letters denote coiled-coil domains. **c** Western blot analysis confirming that the *cti1* CRISPR/Cas9 mutant is a knockout. Chloroplast proteins from wild-type or *cti* mutants were detected using anti-CTI1 antibodies. The asterisk indicates non-specific bands, which were used as protein loading control. The arrow indicates the position of CTI1. Experiments were repeated three times with similar results.

Supplementary Figure 5

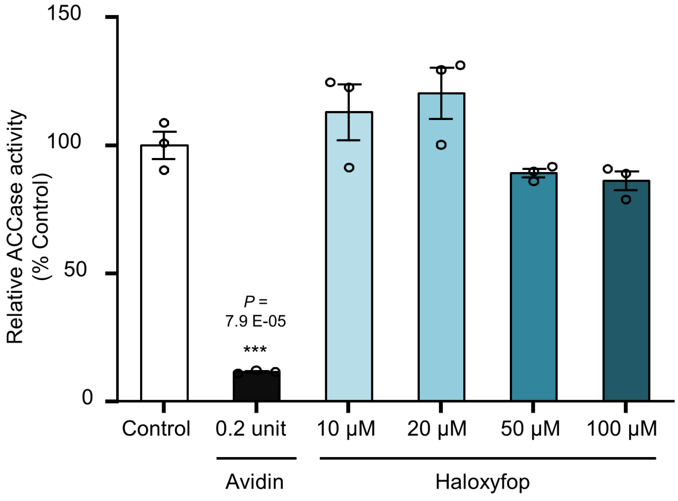

Supplementary Figure 5. ACCase activity in chloroplast crude extracts. The activity, expressed as a percentage of mock control (H_2O), was assayed in chloroplast crude extracts prepared from Arabidopsis leaf tissues in the presence of 0.2-units of the general ACCase inhibitor avidin, or in the presence of increasing concentrations of haloxyfop, a specific inhibitor of homomeric ACCase. Data are means ± SEM, n = 3 individual biological replicates. Asterisks indicate significant difference from the control as determined by a two-tailed Student's *t* test at ***P < 0.001.

Supplementary Figure 6

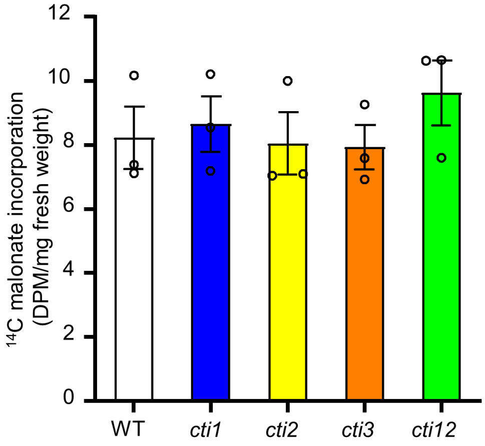

Supplementary Figure 6. ^{14}C-malonate incorporation in rosette leaves of different *cti* mutants. The rates of malonate incorporation were measured by quantifying ^{14}C-malonate into total lipids of leaves from 4-week old plants. Data are means ± SEM, n = 3 biologically independent plants.

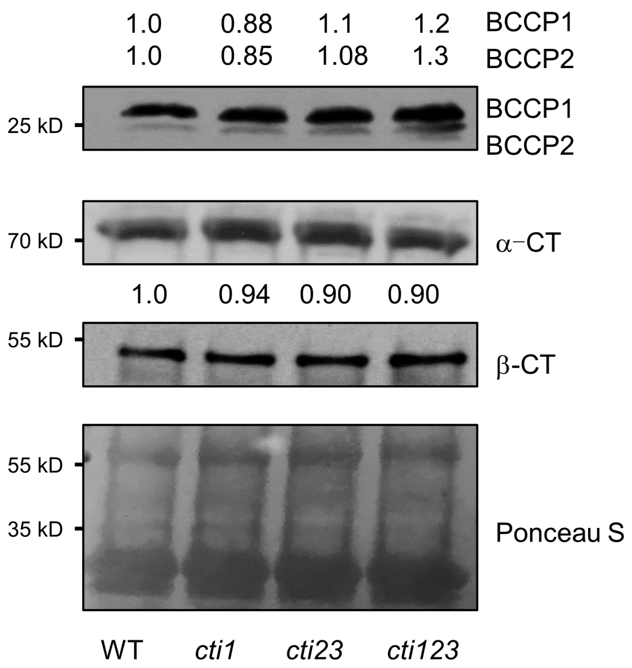

Supplementary Figure 7. Comparison of the abundance of htACCase subunits in wild-type and *cti* mutant plants. Protein extracts from 4-week old plant leaves were used for this experiment. Each subunit was detected using a specific antibody, with the exception of BCCP1 and BCCP2, that were both detected with the same antibody. The relative protein abundance of the different proteins is indicated above the pictures. The relative protein level for each subunit was normalized to that in WT, which was set to 1. ImageJ was used to quantify relative protein abundance. The results shown are representative of three biologically independent samples.

Supplementary Figure 8

a

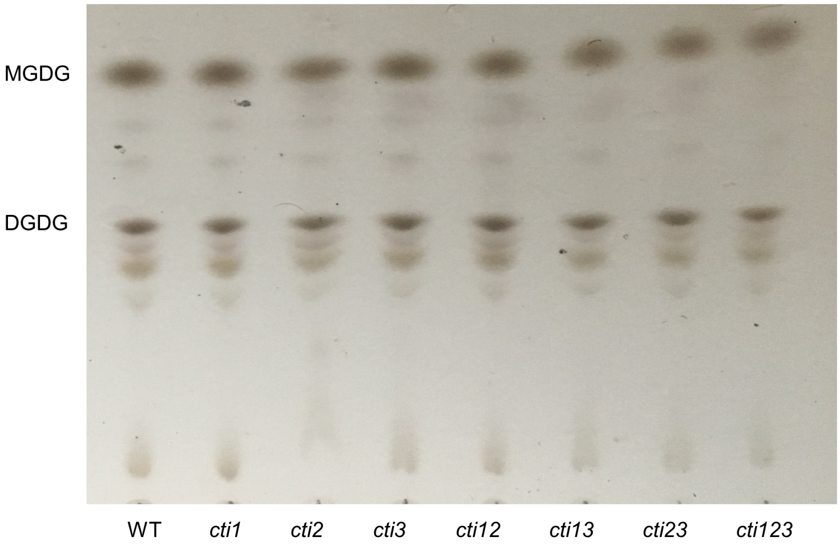

b

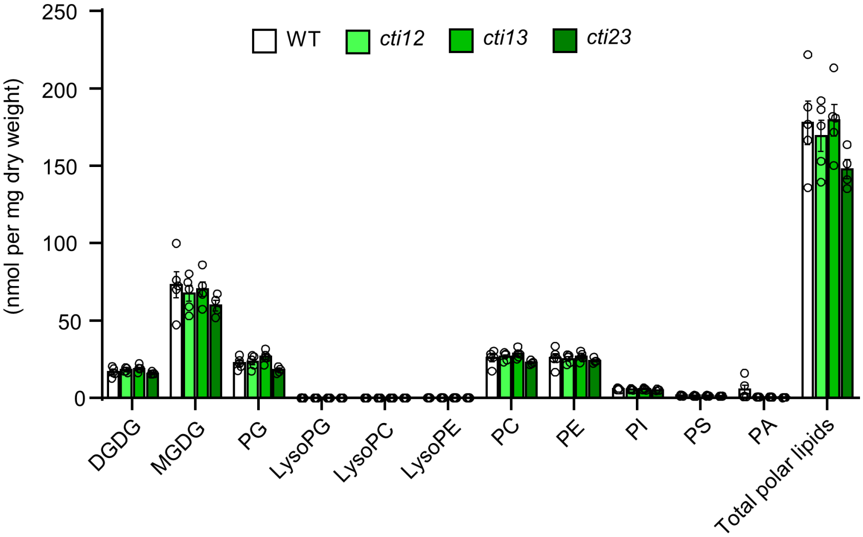

Supplementary Figure 8. Polar lipid composition of Arabidopsis leaf tissues in different genotypes. a Separation of polar lipids by thin layer chromatography. DGDG, digalactosyldiacylglycerol; MGDG, monogalactosyldiacylglycerol. Experiments were repeated three times with similar results. **b** Lipidomic analysis of polar lipids by triple quadrupole mass spectrometer. Plants were grown in long-day condition for 4 weeks, and leaf samples were used for lipid quantification. Data are means ± SEM, n = 5 biologically independent plants.

Supplementary Figure 9

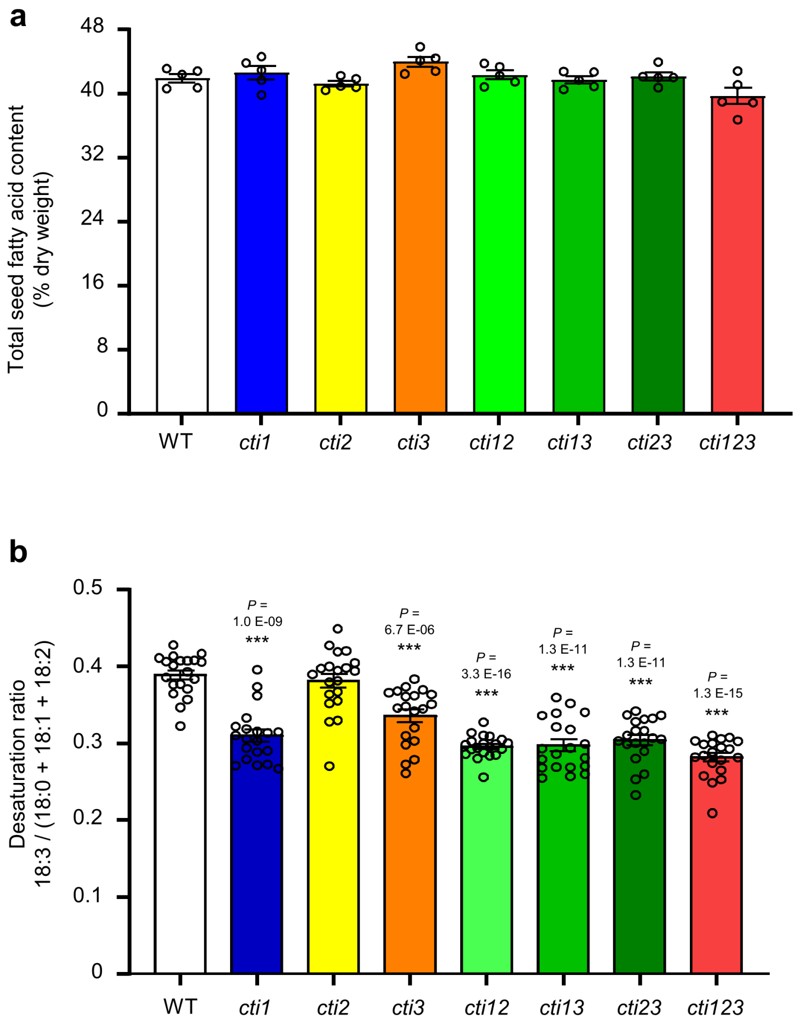

Supplementary Figure 9. Seed phenotype of *cti* mutants. a Total fatty acid content of mature dry seeds, as determined by gas chromatography and expressed on a dry weight basis. Data are means ± SEM, scatterplots show individual plant replicates as circles. **b** Desaturation ratio of C18 fatty acids in *cti* mutant seeds. Data are means ± SEM, scatterplots show individual plant replicates as circles. Asterisks indicate significant difference from the wild-type control as determined by a two-tailed Student's *t* test at ***P < 0.001.

Supplementary Figure 10

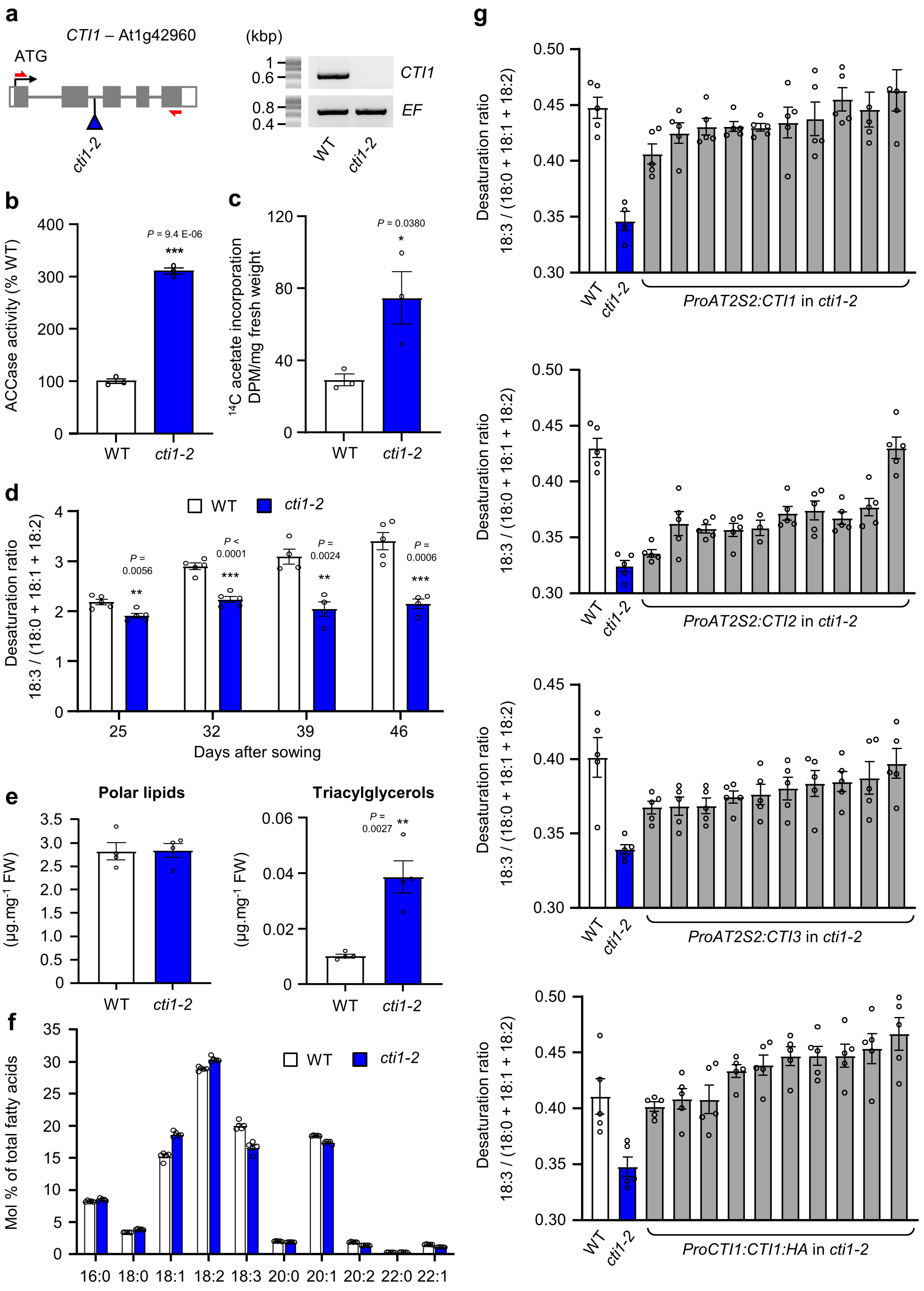

Supplementary Figure 10. Characterization of a *cti1* T-DNA knockout mutant. **a** Molecular characterization of *cti1-2*. The structure of the *CTI1* gene showing the position of the T-DNA insertion in *cti-2* is presented. The confirmed flanking sequence tag is anchored in the gene structure and represented by a vertical bar. Closed boxes represent exons and open boxes untranslated regions. Accumulation of *CTI1* mRNA in wild-type and transgenic backgrounds was studied by RT-PCR on 3-week old rosette leaves. *EF1αA4* (*EF*) gene expression was used as a constitutive control. Primers used for this study are indicated as arrows. The experiment was repeated three times with similar results. **b** ACCase activity in *cti1-2* leaves compared to wild-type leaves. Protein extracts from soluble fractions of 4-week old leaves were used for measuring ACCase activity. Data are means ± SEM, n = 3 biologically replicates. Asterisks indicate significant difference from the wild-type control as determined by a two-tailed Student's *t* test at ***P < 0.001. **c** Enhanced rate of fatty acid synthesis in 4-week old leaves of *cti1-2* as revealed by ^{14}C-acetate labeling. Data are means ± SEM, n = 3 biologically replicates. Asterisks indicate significant difference from the wild-type control as determined by a two-tailed Student's *t* test at *P<0.05. **d** Decreased C18 fatty acid desaturation ratio in *cti1-2* leaves compared to wild-type leaves. The total fatty acid composition of leaves harvested between 25 and 46 days after sowing was determined by gas chromatography. Data are means ± SEM, n = 4 individual plant replicates. Asterisks indicate significant difference from the wild-type control as determined by a two-tailed Student's *t* test at ***P < 0.001 and **P < 0.01, respectively. **e** Polar lipid and triacylglycerol contents in *cti1-2* mutant leaves compared to that of wild-type plants. Total lipids extracted from leaves were separated by thin-layer chromatography. Polar lipids and triacylglycerol were further analyzed by gas chromatography. Data are means ± SEM, n = 4 individual plant replicates. Asterisks indicate significant difference from the wild-type control as determined by a two-tailed Student's *t* test at **P < 0.01. The experiment was repeated twice with similar results. **f** Fatty acid composition of *cti1-2* and wild-type mature dry seeds. Total fatty acids were analyzed by gas chromatography on batches of 20 seeds from different plants. Data are means ± SEM, n = 5 individual plant replicates. The experiment was repeated 10 times with similar results. **g** Rescue of the desaturation ratio phenotype among fatty acids of the C18 series in mature *cti1-2* seeds using *ProAT2S2:CTI1*, *ProAT2S2:CTI2*, *ProAT2S2:CTI3*, or *ProCTI1:CTI1:HA* constructs. For each construct-tested, 10 independent transformants were analyzed. The total fatty acid composition of dry seeds was determined by gas chromatography on batches of 20 seeds from different plants. Data are means ± SEM, scatterplots show individual plant replicates as circles.

Supplementary Figure 11

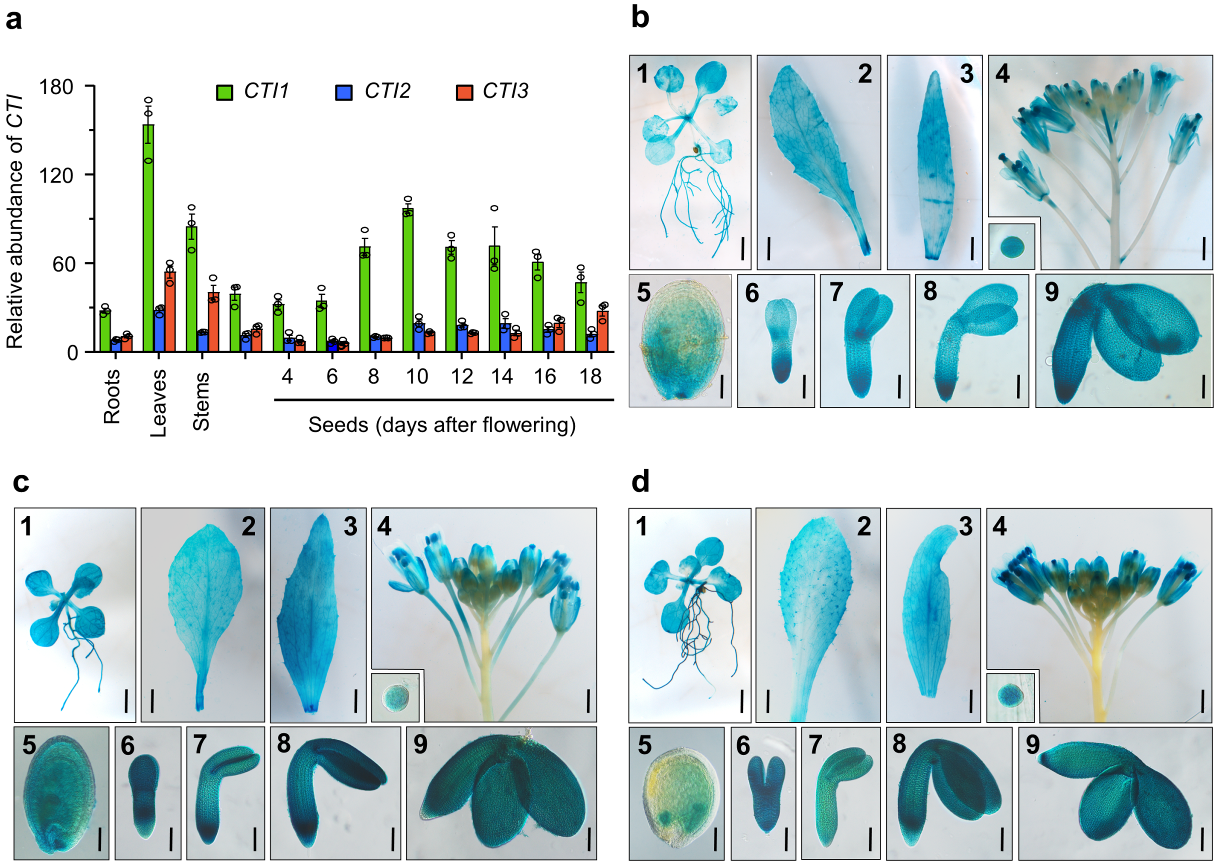

Supplementary Figure 11. Characterization of the expression patterns of *CTI* genes. a qRT-PCR analysis of the expression of *CTI* genes in different organs. The results obtained are standardized to the constitutive *EF1αA4* (*EF*) gene expression level. Values are the means and SEM of three replicates. The three cDNA dilutions used for each organ analyzed were obtained from independent mRNA extractions. **b-d** Pattern of activity of the *ProCTI1:CTI1:uidA* **(b)**, *ProCTI2:CTI2:uidA* **(c),** and *ProCTI3:CTI3:uidA* cassettes **(d)** in transgenic plants. GUS activity was investigated in plantlets (1), in rosette leaves (2), in cauline leaves (3), in flowers (4) and in pollen grains (4, secondary small picture), in seeds (5), in maturing embryos harvested 6 (6), 8(7), 10 (8) or 12 (9) days after anthesis. For histochemical detection of GUS activity, all tissues but embryos were incubated overnight in a staining buffer containing 0.2 mM each of potassium ferrocyanide and potassium ferricyanide. Excised embryos were incubated for 4 h in a buffer containing 2 mM each of potassium ferrocyanide and potassium ferricyanide. The results for GUS activity were observed on whole mounted vegetative organs and inflorescences. The microscopy observations of pollen grains, seeds and excised embryos were performed using Nomarski optics. Bars = 4 mm in (1-4) and 100 µm in (5-9). The experiment was repeated with 18 (*CTI1*), 19 (*CTI2*), and 17 (*CTI3*) independent transformants with similar results. Representative pictures are displayed.

Supplementary Figure 12

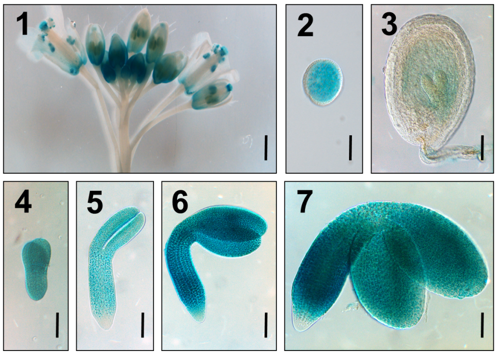

Supplementary Figure 12. The expression pattern of *CTI1*. The pattern of activity of the *ProCTI1(994 bp):uidA* cassette was observed in flowers (1), pollen grains (2), seeds (3), and maturing embryos harvested 6 (4), 8 (5), 10 (6) or 12 (7) days after anthesis. For histochemical detection of GUS activity, all tissues but embryos were incubated overnight in a staining buffer containing 0.2 mM each of potassium ferrocyanide and potassium ferricyanide. Excised embryos were incubated for 4 h in a buffer containing 2 mM each of potassium ferrocyanide and potassium ferricyanide. The results for GUS activity were observed on whole mounted inflorescences. The microscopy observations of pollen grains, seeds and excised embryos were performed using Nomarski optics. Bars = 0.4 mm in (1), 10 µm in (2), and 100 µm in (3-7). The experiment was repeated with 17 independent transformants with similar results. Representative pictures are displayed.

Supplementary Figure 13

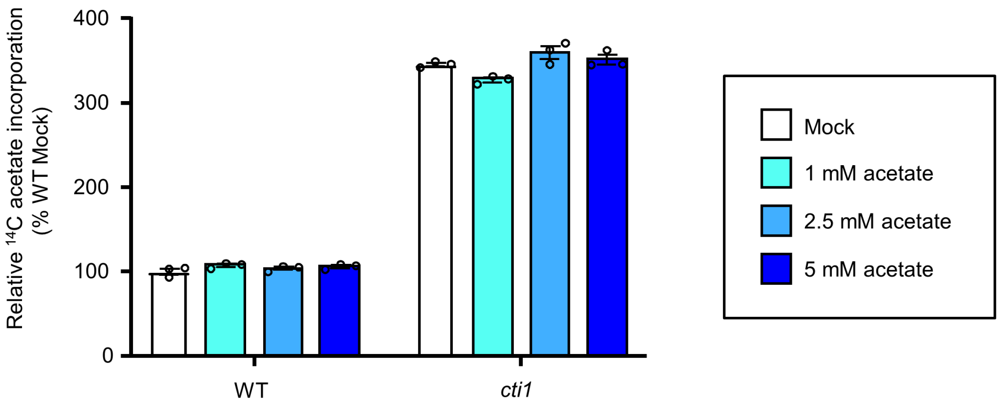

Supplementary Figure 13. ^{14}C-acetate incorporation in different genotypes. The rate of ^{14}C-acetate incorporation into total lipids was measured in leaf tissues from 4-week old plants. Before adding ^{14}C-acetate into the labeling assay, we incubated the plant leaf tissues for 10 minutes in the presence of 1-5 mM unlabeled acetate. Data are means ± SEM, n = 3 biological replicates.

Supplementary Figure 14

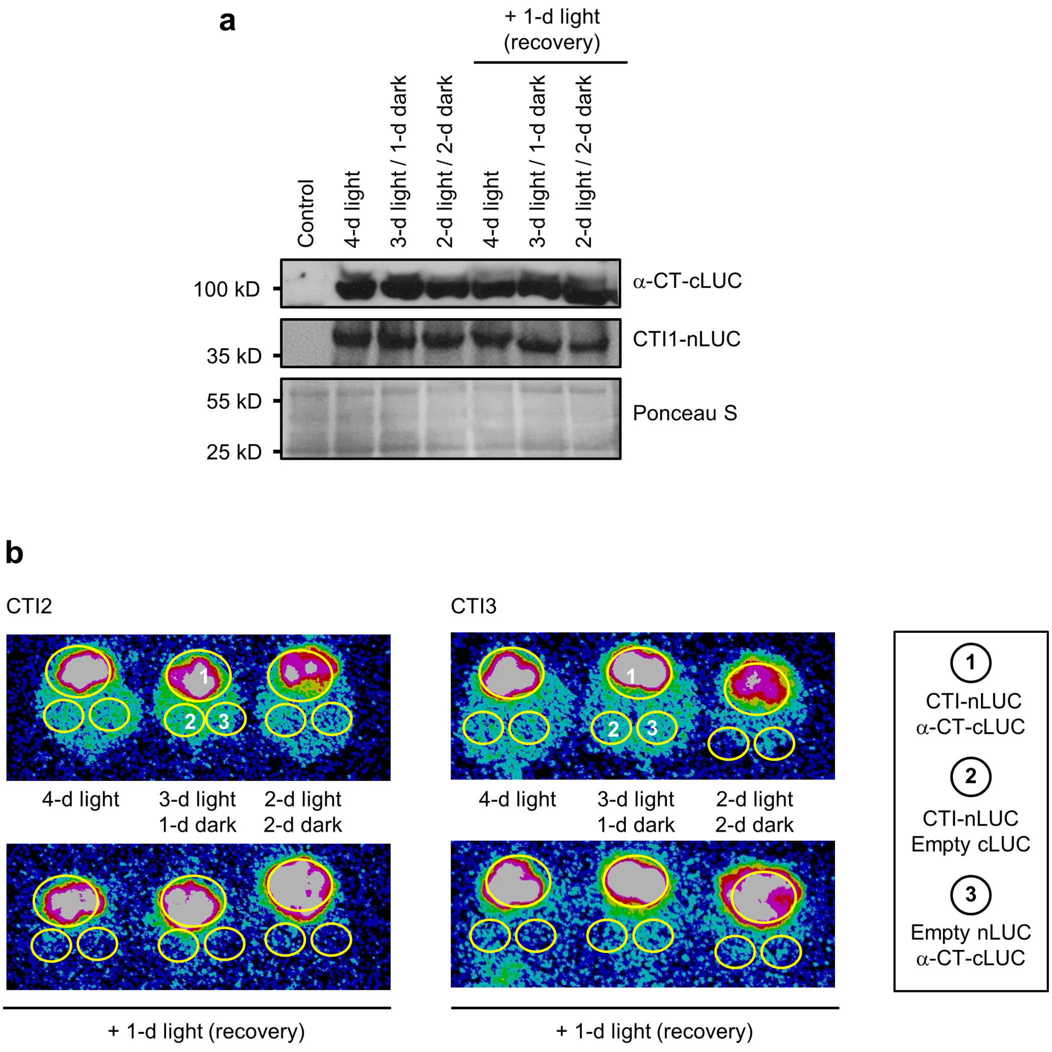

Supplementary Figure 14. Complementary results for the split luciferase assays showing the light dependence of CTI/α-CT interactions. a Determination of protein levels by Western blot, as a control of the experiment displayed in Figure 5e. CTI1 was detected using anti-luciferase antibodies and α-CT was detected using anti-α-CT antibodies. Ponceau S Staining shows protein loading. Experiments were repeated three times with similar results. **b** CTIs interact with α-CT in a light-dependent manner as revealed by split luciferase assays. Split-luciferase assays showed that CTI2 and CTI3 interacted with α-CT in a light-dependent manner. Three combinations of constructs were injected in leaves of *Nicotiana benthamiana.* After transformation, the plants were grown under different light/dark regimes before imaging of luminescence. The results shown are representative of three biologically independent plants.

Supplementary Table 1. Prediction of chloroplast of transit peptides

Name	Length	Score	cTP	CS-score	cTP-length
CTI1	168	0.573	Yes	2.100	59
CTI2	168	0.540	Yes	7.831	53
CTI3	168	0.570	Yes	5.973	56

The chloroplast transit peptides (cTP) were predicted according to http://www.cbs.dtu.dk/services/ChloroP/.

Supplementary Table 2. CTI homologs for phylogenetic analysis

Species	Accession number
Abies pinsapo	GCZN01049633
Abies pinsapo	GCZN01059401
Acrostichum aureum	GEEI01004933
Acrostichum aureum	GEEI01005847
Aegilops tauschii	XP_020163027
Amborella trichopoda	XP_006847741
Amborella trichopoda	XP_011627526
Anabaena sp.	WP_066381195
Ananas comosus	XP_020080497
Ananas comosus	XP_020084289
Ananas comosus	XP_020094682
Ananas comosus	XP_020112360
Ananas comosus	XP_020113051
Aquilegia coerulea	PIA39910
Aquilegia coerulea	PIA46653
Arabidopsis lyrata	XP_020866941
Arabidopsis lyrata	XP_020878203
Arabidopsis lyrata	XP_020886851
Arabidopsis thaliana	AT1G42960
Arabidopsis thaliana	AT3G02900
Arabidopsis thaliana	AT5G16660
Arabis alpina	KFK25887
Arabis alpina	KFK29152
Arabis alpina	KFK37736
Araucaria cunninghamii	GCKF01032890
Asarum sakawanum	TRINITY_DN30450_c0_g1_i1
Asarum sakawanum	TRINITY_DN32083_c0_g1_i1
Asarum sakawanum	TRINITY_DN37551_c1_g1_i1
Asparagus officinalis	XP_020242613
Asparagus officinalis	XP_020248397
Asparagus officinalis	XP_020260129
Asparagus officinalis	XP_020276076
Auxenochlorella protothecoides	XP_011396928
Bathycoccus prasinos	XP_007512887
Beilschmiedia pendula	TRINITY_DN76689_c0_g1_i1
Beilschmiedia pendula	TRINITY_DN93120_c1_g1_i1
Beta vulgaris	XP_010671080
Beta vulgaris	XP_010686851
Brachypodium distachyon	XP_003580468
Brassica rapa	XP_009107643
Brassica rapa	XP_009121465
Brassica rapa	XP_009131552
Bryum argenteum	GCZP01012640
Bryum argenteum	GCZP01036236
Calothrix sp. NIES2098	WP_096588184
Camelina sativa	XP_010420368
Camelina sativa	XP_010453829
Camelina sativa	XP_010461388
Camelina sativa	XP_010463722
Camelina sativa	XP_010478984
Camelina sativa	XP_010485620
Camelina sativa	XP_010492580
Camelina sativa	XP_010500214
Camelina sativa	XP_010503115
Cananga odorata	TRINITY_DN25000_c2_g1_i1
Capsella rubella	XP_006286588
Capsella rubella	XP_006298987
Capsella rubella	XP_023645323

Capsicum annuum	XP_016551797
Capsicum annuum	XP_016564110
Capsicum annuum	XP_016568224
Chlamydomonas reinhardtii	XP_001697999
Chlorella sorokiniana	PRW56266
Cinnamomum longepaniculatum	TRINITY_DN25886_c0_g1_i1
Cinnamomum longepaniculatum	TRINITY_DN33588_c2_g3_i1
Cinnamomum longepaniculatum	TRINITY_DN33915_c3_g4_i1
Coccomyxa subellipsoidea C-169	XP_005648656
Coffea canephora	CDP01771
Coffea canephora	CDP04919
Coffea canephora	CDP07270
Cucumis melo	XP_008453013
Cucumis melo	XP_008459202
Cycas revoluta	GBJU01012216
Cycas revoluta	GBJU01013847
Dactylococcopsis salina	WP_015228729
Elaeis guineensis	XP_010933483
Elaeis guineensis	XP_010935010
Elaeis guineensis	XP_010940244
Elaeis guineensis	XP_010941239
Ephedra trifurca	GBKT01019247
Fischerella muscicola	WP_102205739
Fragaria vesca	XP_004287712
Fragaria vesca	XP_004300120
Fragaria vesca	XP_004307842
Ginkgo biloba	GBI00025979
Ginkgo biloba	GBYR01003250
Glycine max	NP_001237336
Glycine max	NP_001238304
Glycine max	NP_001242410
Glycine max	XP_003517126
Glycine max	XP_003527290
Glycine max	XP_003537695
Glycine max	XP_006572801
Gnetum montanum	GMO00030593
Gossypium raimondii	XP_012434823
Gossypium raimondii	XP_012463460
Gossypium raimondii	XP_012464278
Gossypium raimondii	XP_012472338
Gossypium raimondii	XP_012478175
Helianthus annuus	XP_022008582
Helianthus annuus	XP_022015975
Helianthus annuus	XP_022016260
Helianthus annuus	XP_022038127
Hydrococcus rivularis	WP_073598243
Hymenophyllum caudiculatum	TRINITY_DN1815_c0_g1_i2
Hymenophyllum caudiculatum	TRINITY_DN30536_c0_g1_i1
Jatropha curcas	XP_012065426
Jatropha curcas	XP_012070250
Jatropha curcas	XP_012073609
Klebsormidium nitens	GAQ88763
Lactuca sativa	XP_023748386
Lactuca sativa	XP_023765167
Lactuca sativa	XP_023771370
Lactuca sativa	XP_023772590
Larix kaempferi	JR155991
Larix kaempferi	JR157076
Leptolyngbya frigida	WP_106257279
Lygodium japonicum	FX964017
Lygodium japonicum	FX973459
Manihot esculenta	XP_021601871
Manihot esculenta	XP_021613334

Manihot esculenta	XP_021619384
Manihot esculenta	XP_021622917
Marchantia polymorpha	OAE35691
Medicago truncatula	XP_003603853
Medicago truncatula	XP_003610804
Medicago truncatula	XP_003612406
Microcoleus sp. PCC7113	WP_015185264
Microcystis aeruginosa	WP_002803502
Micromonas pusilla CCMP1545	XP_003061994
Monachosorum maximowiczii	TRINITY_DN15245_c0_g1_i1
Monachosorum maximowiczii	TRINITY_DN66652_c0_g1_i1
Musa acuminata	XP_009386246
Musa acuminata	XP_009397955
Musa acuminata	XP_009408189
Musa acuminata	XP_009415324
Musa acuminata	XP_018678772
Nelumbo nucifera	XP_010266243
Nelumbo nucifera	XP_010270521
Nelumbo nucifera	XP_010275220
Nicotiana attenuata	XP_019235782
Nicotiana attenuata	XP_019244857
Nicotiana attenuata	XP_019253436
Nicotiana attenuata	XP_019263228
Nostoc sp._PCC_7524	WP_015141302
Oryza sativa	ABA99701
Oscillatoria acuminata	WP_015149799
Ostreococcus tauri	XP_003080013
Phalaenopsis equestris	XP_020581270
Phalaenopsis equestris	XP_020598908
Phaseolus vulgaris	XP_007135966
Phaseolus vulgaris	XP_007157084
Phaseolus vulgaris	XP_007158025
Phoenix dactylifera	XP_008786318
Phoenix dactylifera	XP_008788671
Phoenix dactylifera	XP_008799390
Phoenix dactylifera	XP_008812614
Phoenix dactylifera	XP_017698083
Physcomitrella patens	PNR48227
Physcomitrella patens	XP_001777705
Picea sitchensis	ABK21292
Picea sitchensis	ABK21824
Pinus pinaster	sp_v3.0_unigene17583
Piper betle	TRINITY_DN11695_c0_g1_i2
Piper betle	TRINITY_DN12282_c0_g2_i1
Piper betle	TRINITY_DN9668_c0_g1_i1
Populus trichocarpa	XP_002307807
Populus trichocarpa	XP_002310110
Populus trichocarpa	XP_002319245
Populus trichocarpa	XP_002325872
Prunus avium	XP_021804504
Prunus avium	XP_021816205
Prunus avium	XP_021827567
Pseudanabaena biceps PCC7429	ELS34401
Pseudotsuga menziesii	GFFY01023377
Pseudotsuga menziesii	GFFY01046732
Pteridium aquilinum	GASP01016278
Ricinus communis	XP_002510707
Ricinus communis	XP_002520082
Ricinus communis	XP_002521740
Ricinus communis	XP_015576339
Sarcandra glabra	TRINITY_DN16989_c0_g1_i1
Sarcandra glabra	TRINITY_DN9024_c0_g1_i1
Sarcandra glabra	TRINITY_DN9829_c0_g1_i1

Schisandra chinensis	TRINITY_DN210599_c0_g1_i1
Schisandra chinensis	TRINITY_DN212746_c2_g1_i1
Selaginella moellendorffii	XP_002962472
Selaginella moellendorffii	XP_002962810
Solanum lycopersicum	NP_001315610
Solanum lycopersicum	XP_004238447
Solanum lycopersicum	XP_004253123
Sorghum bicolor	XP_002448424
Spinacia oleracea	XP_021851018
Spinacia oleracea	XP_021853631
Tarenaya hassleriana	XP_010525058
Tarenaya hassleriana	XP_010530267
Tarenaya hassleriana	XP_010539029
Tarenaya hassleriana	XP_010549854
Tarenaya hassleriana	XP_019056670
Taxus baccata	TBA00007052
Triticum urartu	EMS65734
Vigna angularis	XP_017407912
Vigna angularis	XP_017426848
Vigna angularis	XP_017434336
Vitis vinifera	XP_002270877
Vitis vinifera	XP_002273917
Vitis vinifera	XP_002276709
Warburgia ugandensis	TRINITY_DN51967_c3_g1_i3
Warburgia ugandensis	TRINITY_DN55845_c0_g1_i5
Zostera marina	KMZ66544
Zostera marina	KMZ69807
Zostera marina	KMZ72120

Supplementary Table 3. Primers used for construct preparation

Construct	Sequence cloned	Primer	Primer sequence (5'→3')
ProCTI1:CTI1:uidA	*At1g42960 (2,035-bp promoter sequence + gene without STOP)*	Forward	GGGGACAAGTTTGTACAAAAAAGCAGGCTTCCTAGTGCAACGCTTTTC
		Reverse	GGGGACCACTTTGTACAAGAAAGCTGGGTCGGCTGTAGCCTCGATTTC
ProCTI1:uidA	*At1g42960 (994-bp promoter sequence)*	Forward	GGGGACAAGTTTGTACAAAAAAGCAGGCTTCCTTTTTTGGTGAAAATTTACG
		Reverse	GGGGACCACTTTGTACAAGAAAGCTGGGTCGGATGGAAGAAACTGTGTC
ProCTI3:CTI3:uidA	*At5g16660 (2,035-bp promoter sequence + gene without STOP)*	Forward	GGGGACAAGTTTGTACAAAAAAGCAGGCTTCTTTGCATCCAACCAGC
		Reverse	GGGGACCACTTTGTACAAGAAAGCTGGGTCAGTCGTAGCTTCAACCTC
ProCTI2:CTI2:uidA	*At3g02900 (1,981-bp promoter sequence + gene without STOP)*	Forward	GGGGACAAGTTTGTACAAAAAAGCAGGCTTCAAAAAGTCACCTTGAATGAAG
		Reverse	GGGGACCACTTTGTACAAGAAAGCTGGGTCTGCAGTAGCTTCTGC
ProAT2S2:CTI1	*At1g42960 (cDNA)*	Forward	GGGGACAAGTTTGTACAAAAAAGCAGGCTTCATGGCGTCTCTTTCTTCTAC
		Reverse	GGGGACCACTTTGTACAAGAAAGCTGGGTCTCAATTTCAAATCCTAAAAACA G
ProAT2S2:CTI2	*At3g02900 (cDNA)*	Forward	GGGGACAAGTTTGTACAAAAAAGCAGGCTTCATGGCGTCCTTGGTAG
		Reverse	GGGGACCACTTTGTACAAGAAAGCTGGGTCTGGAGCAGAGCATTACATTC
ProAT2S2:CTI3	*At5g16660 (cDNA)*	Forward	GGGGACAAGTTTGTACAAAAAAGCAGGCTTCATGGCGTCGTGTATTG
		Reverse	GGGGACCACTTTGTACAAGAAAGCTGGGTCTCAAGTCGTAGCTTCAACC
Pro35S:CTI1:HA	*At1g42960 (cDNA without STOP codon)*	Forward	GGGGACAAGTTTGTACAAAAAAGCAGGCTTCATGGCGTCTCTTTCTTCTAC
		Reverse	GGGGACCACTTTGTACAAGAAAGCTGGGTCGGCTGTAGCCTCGATTTC
ProCTI1:CTI1:HA	*At1g42960 (994-bp promoter sequence + gene without STOP)*	Forward	GGGGACAAGTTTGTACAAAAAAGCAGGCTTCCTTTTTTGGTGAAAATTTACG
		Reverse	GGTAAAAGATGTTAATTAACCCAGCTCCACCTCCACCTCCAG
	3xHA cDNA	Forward	CTGGAGGTGGAGGTGGAGCTGGGTTAATTAACATCTTTTACC
		Reverse	GGGGACCACTTTGTACAAGAAAGCTGGGTCTCAGCTGCACTGAGCAGCGTA A
ProCTI1:HIS	*At1g42960 (500-bp promoter sequence)*	Forward	GGAATTCCTGGTTGTAAAATTTTGAAGATAC
		Reverse	TCCCCGCGGGGAGGATGGAAGAAACTGTGTC
ProYAO:CTI1:Cas9	*AT1g42960 primers*	Forward	GATTTGGTGCATATACACATCCGA
		Reverse	AAACTCGGATGTGTATATGCACCA
ProYAO:CTI2:Cas9	*At3g02900 primers*	Forward	GATTAGGCCTTCGTCATAGTACAT
		Reverse	AAACATGTACTATGACGAAGGCCT
ProYAO:CTI3:Cas9	*At5g16660 primers*	Forward	GATTTGATAAGTGTTCTTTAGTTT
		Reverse	AAACAAACTAAAGAACACTTATCA
pENTR/D-TOPO-CTI1	*At1g42960 (cDNA)*	Forward	CACCATGGCGTCTCTTTCTTCTACCT
		Reverse	GGCTGTAGCCTCGATTTCAT
pENTR/D-TOPO-CTI2	*At3g02900 (cDNA)*	Forward	CACCATGTGTTCGTCTTCCTCTTGTATG

		Reverse	TGCAGTAGCTTCTGCTTCTGC
pENTR/D-TOPO-CTI3	*At5g16660 (cDNA)*	Forward	CACCATGGCGTCGTGTATTGCTACTG
		Reverse	AGTCGTAGCTTCAACCTCTGGG
pENTR/D-TOPO-α-CT	*AT2G38040 codon-optimized sequence*	Forward	CACCATGGCTTCTATCTCACACTCTAGTT
		Reverse	AGCGAAACTAGGATTCACCAT
pENTR/D-TOPO-TGD2	*AT3G20320 (cDNA)*	Forward	CACCATGATTGGGAATCCAGTAAT
		Reverse	TAGTAGCCTGCTTAGGGATTTGA
pENTR/D-TOPO-CTI1 (coiled-coil)	*At1g42960 (cDNA)*	Forward	CACCATGCCACAGATCAGCAAG
		Reverse	GGCTGTAGCCTCGATTTCAT
pENTR/D-TOPO-CTI2 (coiled-coil)	*At3g02900 (cDNA)*	Forward	CACCATGATCCGAAGATCAGTGCTGAG
		Reverse	TGCAGTAGCTTCTGCTTCT
pENTR/D-TOPO-CTI3 (coiled-coil)	*At5g16660 (cDNA)*	Forward	CACCATGTTTGCTCCACAGATCCGAA
		Reverse	AGTCGTAGCTTCAACCTCTG
pENTR/D-TOPO-α-CT (coiled-coil)	*AT2G38040 codon-optimized sequence*	Forward	CACCATGATCAACATGAAGAAGAGAGAGG
		Reverse	AGCGAAACTAGGATTCACCAT

Note that bases in red correspond to restriction enzyme recognition sites, while bases in blue correspond to BP recombination sequences.

Supplementary Table 4. Primers used for the molecular characterization of the mutants (as displayed in Supplemental Figures 11A).

Mutant	AGI	Forward primer (5'→3')	Reverse primer (5'→3')
EF1αA4	At5g60390	ATGCCCCAGGACATCGTGATTTCAT	TTGGCGGCACCCTTAGCTGGATCA
CTI1	At1g42960	ATGGCGTCTCTTTCTTCTAC	TCAATTTCAAATCCTAAAAACAG

Supplementary Table 5. Primers used for quantitative RT-PCR

Gene	AGI	Forward primer (5'→3')	Reverse primer (5'→3')
EF1alphaA4	At5g60390	CTGGAGGTTTTGAGGCTGGTAT	CCAAGGGTGAAAGCAAGAAGA
CTI1	At1g42960	GCTCGTTGTGTCAGTGTTTCATTTGG	GCACCGACTATAAGTCCGCCC
CTI2	At3g02900	TTTGCTCCACAGATCCGAAGATCAGT	CGACGAAACCTTGTCAATGGCG
CTI3	At5g16660	AGAAGCCGAAACAGCCAACG	AGCTTCAACCTCTGGGTCAGT

Supplementary Table 6. Primers used for yeast-2-hybrid

	Forward primer (5'→3')
T7	TAATACGACTCACTATAGGG
3'AD	AGATGGTGCACGATGCACAG
