# Supplementary material for: Docking of acetyl-CoA carboxylase to the plastid envelope membrane attenuates fatty acid production in plants
Source: Nat Commun. 2020 Dec 3;11:6191. doi: 10.1038/s41467-020-20014-5 (PMC7712654; doi:10.1038/s41467-020-20014-5)
Supplement: Supplementary file 2 — Reporting Summary [file 41467_2020_20014_MOESM2_ESM.pdf]

nature research

Corresponding author(s): Jay Thelen

Last updated by author(s): Oct 21, 2020

Reporting Summary

Nature Research wishes to improve the reproducibility of the work that we publish. This form provides structure for consistency and transparency in reporting. For further information on Nature Research policies, see our Editorial Policies and the Editorial Policy Checklist.

Statistics

For all statistical analyses, confirm that the following items are present in the figure legend, table legend, main text, or Methods section.

n/a	Confirmed	
☐	☒	The exact sample size (n) for each experimental group/condition, given as a discrete number and unit of measurement
☐	☒	A statement on whether measurements were taken from distinct samples or whether the same sample was measured repeatedly
☐	☒	The statistical test(s) used AND whether they are one- or two-sided<br>*Only common tests should be described solely by name; describe more complex techniques in the Methods section.*
☒	☐	A description of all covariates tested
☒	☐	A description of any assumptions or corrections, such as tests of normality and adjustment for multiple comparisons
☐	☒	A full description of the statistical parameters including central tendency (e.g. means) or other basic estimates (e.g. regression coefficient) AND variation (e.g. standard deviation) or associated estimates of uncertainty (e.g. confidence intervals)
☐	☒	For null hypothesis testing, the test statistic (e.g. F, t, r) with confidence intervals, effect sizes, degrees of freedom and P value noted<br>*Give P values as exact values whenever suitable.*
☒	☐	For Bayesian analysis, information on the choice of priors and Markov chain Monte Carlo settings
☒	☐	For hierarchical and complex designs, identification of the appropriate level for tests and full reporting of outcomes
☒	☐	Estimates of effect sizes (e.g. Cohen's d, Pearson's r), indicating how they were calculated

Our web collection on statistics for biologists contains articles on many of the points above.

Software and code

Policy information about availability of computer code

Data collection	CRISPR-P v2.0 for guide RNA design.
Data analysis	Microsoft Excel 2016, Graphpad Prism 8.0.1 for statistics and bar graphs, MO.Offinity Analysis (X86) for MST analysis

For manuscripts utilizing custom algorithms or software that are central to the research but not yet described in published literature, software must be made available to editors and reviewers. We strongly encourage code deposition in a community repository (e.g. GitHub). See the Nature Research guidelines for submitting code & software for further information.

Data

Policy information about availability of data

All manuscripts must include a data availability statement. This statement should provide the following information, where applicable:

- Accession codes, unique identifiers, or web links for publicly available datasets
- A list of figures that have associated raw data
- A description of any restrictions on data availability

Source data are provided with this paper as Source Data file. Other data and biological materials are available from the corresponding other upon reasonable request.

Field-specific reporting

Life sciences study design

All studies must disclose on these points even when the disclosure is negative.

Sample size	The sample size was chosen on the basis of prior studies and showed significant with similar sample sizes (for example see Bates et al., 2014 PNAS, https://doi.org/10.1073/pnas.1318511111; Salie et al., 2016 Plant Cell, https://doi.org/10.1105/tpc.16.00317; Ye et al., 2020 Journal of Biological Chemistry, https://doi: 10.1074/jbc.RA120.012877; Baud et al., 2010 Plant Journal, https://doi.org/10.1111 j.1365-313X.2010.04332.x).
Data exclusions	Some gas chromtography (GC) analyses were excluded due to the leakage of sample vials, leading to no peaks captured by the GC.
Replication	For RT-qPCR analyses, single plant cultures were realized and the replications consisted in the analysis of three different plants for each genotype.<br>For yeast one hybrid assays, the replications consisted in the study of five independent transformants for each combination of constructs tested.<br>Fore lipidomics, five individual plants were set as five replicates.<br>All the other experiments were repeated with similar results (for details see legends of the figures and source data with reproductivity details).
Randomization	For the culture of Arabidopsis plants, the location of the different genotypes on the trays were randomized. For the harvest of plant tissues (e.g. rosette leaves, seeds, or embryos), protoplast cells, bacterial and yeast strains, the samples or clones were randomly selected throughout this study.
Blinding	For the culture of Arabidopsis lines, the plants were numbered to blind the genotypes. For the other experiments, the investigator was not blind.

Reporting for specific materials, systems and methods

We require information from authors about some types of materials, experimental systems and methods used in many studies. Here, indicate whether each material, system or method listed is relevant to your study. If you are not sure if a list item applies to your research, read the appropriate section before selecting a response.

Materials & experimental systems

n/a	Involved in the study	
☐	☒	Antibodies
☒	☐	Eukaryotic cell lines
☒	☐	Palaeontology and archaeology
☒	☐	Animals and other organisms
☒	☐	Human research participants
☒	☐	Clinical data
☒	☐	Dual use research of concern

Methods

n/a	Involved in the study	
☒	☐	ChIP-seq
☒	☐	Flow cytometry
☒	☐	MRI-based neuroimaging

Antibodies

Antibodies used	Anti-CTI1<br>Anti-E37<br>Anti-a-CT<br>Anti-BCCPs<br>Anti-b-CT<br>Anti-HA, Roche, Clone 12CA5,REF 11583816001<br>Anti-Myc, Millpore, Clone 4A6, Cat # 05-724
Validation	Anti-MYC and Anti-HA antibodies were validated by the manufacturers. Anti-E37 antibody was validated in various dicots and monocots by Teyssier et al. (The Plant Journal 1996, https://doi.org/10.1046/j.1365-313X.1996.10050903.x) and in Arabidopsis thaliana by Baud et al. (The Plant Journal 2010, https://doi.org/10.1111/j.1365-313X.2010.04332.x). Anti-a-CT, Anti-BCCPs, Anti-b-CT antibodies were validated as described in Thelen et al. (Arch. Biochem. Biophys. 2002, https://doi: 10.1006/abbi.1998.0900.). Anti-CTI1 was validated in Arabidopsis in this study, in which negative controls were used (Supplementary Figure 4c).
